# Imaging biomarker roadmap for cancer studies

**DOI:** 10.1038/nrclinonc.2016.162

**Published:** 2016-10-11

**Authors:** James P. B. O’Connor, Eric O. Aboagye, Judith E. Adams, Hugo J. W. L. Aerts, Sally F. Barrington, Ambros J. Beer, Ronald Boellaard, Sarah E. Bohndiek, Michael Brady, Gina Brown, David L. Buckley, Thomas L. Chenevert, Laurence P. Clarke, Sandra Collette, Gary J. Cook, Nandita M. deSouza, John C. Dickson, Caroline Dive, Jeffrey L. Evelhoch, Corinne Faivre-Finn, Ferdia A. Gallagher, Fiona J. Gilbert, Robert J. Gillies, Vicky Goh, John R. Griffiths, Ashley M. Groves, Steve Halligan, Adrian L. Harris, David J. Hawkes, Otto S. Hoekstra, Erich P. Huang, Brian F. Hutton, Edward F. Jackson, Gordon C. Jayson, Andrew Jones, Dow-Mu Koh, Denis Lacombe, Philippe Lambin, Nathalie Lassau, Martin O. Leach, Ting-Yim Lee, Edward L. Leen, Jason S. Lewis, Yan Liu, Mark F. Lythgoe, Prakash Manoharan, Ross J. Maxwell, Kenneth A. Miles, Bruno Morgan, Steve Morris, Tony Ng, Anwar R. Padhani, Geoff J. M. Parker, Mike Partridge, Arvind P. Pathak, Andrew C. Peet, Shonit Punwani, Andrew R. Reynolds, Simon P. Robinson, Lalitha K. Shankar, Ricky A. Sharma, Dmitry Soloviev, Sigrid Stroobants, Daniel C. Sullivan, Stuart A. Taylor, Paul S. Tofts, Gillian M. Tozer, Marcel van Herk, Simon Walker-Samuel, James Wason, Kaye J. Williams, Paul Workman, Thomas E. Yankeelov, Kevin M. Brindle, Lisa M. McShane, Alan Jackson, John C. Waterton

**Affiliations:** 1 CRUK and EPSRC Cancer Imaging Centre in Cambridge and Manchester, University of Manchester, Manchester, UK; 2 Department of Surgery and Cancer, Imperial College, London, UK; 3 Department of Clinical Radiology, Central Manchester University Hospitals NHS Foundation Trust, Manchester, UK; 4 Department of Radiation Oncology, Harvard Medical School, Boston, MA; 5 CRUK and EPSRC Comprehensive Imaging Centre at KCL and UCL, Kings College London, London, UK; 6 Department of Nuclear Medicine, University Hospital Ulm, Ulm, Germany; 7 Department of Nuclear Medicine and Molecular Imaging, University Medical Center Groningen, Groningen, The Netherlands; 8 CRUK and EPSRC Cancer Imaging Centre in Cambridge and Manchester, University of Cambridge, Cambridge, UK; 9 CRUK and EPSRC Cancer Imaging Centre, University of Oxford, Oxford, UK; 10 Radiology Department, Royal Marsden Hospital, London, UK; 11 Division of Biomedical Imaging, University of Leeds, Leeds, UK; 12 Department of Radiology, University of Michigan, Ann Arbor, MI; 13 Cancer Imaging Program, National Cancer Institute, Bethesda, MD; 14 Biostatistics, EORTC, Brussels, Belgium; 15 CRUK Cancer Imaging Centre, The Institute of Cancer Research, London, UK; 16 CRUK and EPSRC Cancer Imaging Centre at KCL and UCL, University College London, London, UK; 17 Clinical and Experimental Pharmacology, CRUK Manchester Institute, Manchester, UK; 18 Translational Biomarkers, Merck & Co., Inc, West Point, PA; 19 Radiotherapy Related Research Group, University of Manchester, Manchester, UK; 20 Cancer Imaging and Metabolism, Moffitt Cancer Center, Tampa, FL; 21 Department of Radiology and Nuclear Medicine, VU University Medical Centre, Amsterdam, The Netherlands; 22 Biometric Research Program, National Cancer Institute, Bethesda, MD; 23 Department of Medical Physics, University of Wisconsin, Madison, WI; 24 Institute of Cancer Sciences, University of Manchester, Manchester, UK; 25 Medical Physics, The Christie Hospital NHS Foundation Trust, Manchester, UK; 26 EORTC Headquarters, EORTC, Brussels, Belgium; 27 Department of Radiation Oncology, University of Maastricht, Maastricht, Netherlands; 28 Department of Imaging, Gustave Roussy Cancer Campus, Villejuif, France; 29 Imaging Research Labs, Robarts Research Institute, London, Ontario, Canada; 30 Department of Radiology, Memorial Sloan Kettering Cancer Center, New York, NY; 31 Centre for Advanced Biomedical Imaging, University College London, London, UK; 32 Northern Institute for Cancer Research, Newcastle University, Newcastle, UK; 33 Cancer Studies and Molecular Medicine, University of Leicester, Leicester, UK; 34 Institute of Epidemiology and Health, University College London, London, UK; 35 Paul Strickland Scanner Centre, Mount Vernon Hospital, London, UK; 36 Department of Radiology, The Johns Hopkins University School of Medicine, Baltimore, MD; 37 Institute of Cancer and Genomics, University of Birmingham, Birmingham, UK; 38 Breakthrough Breast Cancer Research Centre, The Institute of Cancer Research, London, UK; 39 Molecular Imaging Center Antwerp, University of Antwerp, Antwerp, Belgium; 40 Department of Radiology, Duke University School of Medicine, Durham, NC; 41 Brighton and Sussex Medical School, University of Sussex, Brighton, UK; 42 Department of Oncology and Metabolism, University of Sheffield, Sheffield, UK; 43 MRC Biostatistics Unit, Cambridge, UK; 44 CRUK Cancer Therapeutics Unit, The Institute of Cancer Research, London, UK; 45 Institute of Computational Engineering and Sciences, The University of Texas, Austin, TX

## Abstract

Imaging biomarkers (IBs) are integral to the routine management of patients with cancer. IBs used daily in oncology include clinical TNM stage, objective response and left ventricular ejection fraction. Other CT, MRI, PET and ultrasonography biomarkers are used extensively in cancer research and drug development. New IBs need to be established either as useful tools for testing research hypotheses in clinical trials and research studies, or as clinical decision-making tools for use in healthcare, by crossing ‘translational gaps’ through validation and qualification. Important differences exist between IBs and biospecimen-derived biomarkers and, therefore, the development of IBs requires a tailored ‘roadmap’. Recognizing this need, Cancer Research UK (CRUK) and the European Organisation for Research and Treatment of Cancer (EORTC) assembled experts to review, debate and summarize the challenges of IB validation and qualification. This consensus group has produced 14 key recommendations for accelerating the clinical translation of IBs, which highlight the role of parallel (rather than sequential) tracks of technical (assay) validation, biological/clinical validation and assessment of cost-effectiveness; the need for IB standardization and accreditation systems; the need to continually revisit IB precision; an alternative framework for biological/clinical validation of IBs; and the essential requirements for multicentre studies to qualify IBs for clinical use.

A biomarker is a “defined characteristic that is measured as an indicator of normal biological processes, pathogenic processes or responses to an exposure or intervention, including therapeutic interventions” (REFS 1,^2^). The current FDA–NIH Biomarker Working Group definition — adopted in this consensus statement — states explicitly that “molecular, histologic, radiographic or physiologic characteristics are examples of biomarkers” (REF. 2). This approach seeks to clarify inconsistency in terminology, because some previous definitions have restricted the scope of biomarkers to describing biological molecules. Such narrow definitions regard values obtained from imaging and other techniques as measurements of an underlying biomarker, rather than being biomarkers themselves. However, the current FDA–NIH definition takes a broader view. Earlier definitions also stated that biomarkers should have ‘putative’ diagnostic or prognostic use^3^, although this requirement is no longer specified by FDA/NIH. Biomarkers must be measured, but can be numerical (quantitative) or categorical (either a quantitative value or qualitative data; for definitions, see Supplementary information S1 (table)).

The use of both imaging biomarkers (IBs) and biospecimen-derived biomarkers is widespread in oncology. In healthcare settings, biomarker uses include screening for disease; diagnosing and staging cancer; targeting surgical and radiotherapy treatments; guiding patient stratification; and predicting and monitoring therapeutic efficacy, and/or toxicity^4^. In research, biomarkers guide the development of investigational drugs as they progress along the pharmacological audit trail^5^, in which they can indicate the presence of drug targets, target inhibition, biochemical pathway modulation or pathophysiological alteration by investigational drugs; drug therapeutic efficacy in specific groups of patients; and tracking of drug resistance^6^. The use of biomarkers has led to the identification of potentially successful drugs early in the developmental pipeline, thereby accelerating market approval for some therapies and enabling drug developers to reduce overall costs by identifying ineffective or toxic compounds at the earliest opportunity^5^.

Despite some biomarkers being used extensively and others showing great potential^7^,^8^, a surprisingly limited number of biomarkers guide clinical decisions^9^–^11^. Some putative cancer biomarkers are not adopted because they do not measure a relevant biological feature nor enable disease diagnosis or outcome prediction. In such cases, the biomarker is appropriately devalidated^12^. Many other promising biomarkers, however, are neither devalidated nor qualified for use in research or healthcare settings and, instead, are confined in the academic literature without real application owing to a lack of efficient and effective strategies for biomarker translation^13^.

All biomarkers, including IBs, must cross two ‘translational gaps’ before they can be used to guide clinical decisions^14^,^15^ (FIG. 1). Biomarkers that can reliably be used to test medical hypotheses cross the first gap becoming useful ‘medical research tools’; if the biomarker crosses the second gap then it becomes a ‘clinical decision-making tool’. Some biomarkers that have only crossed the first translational gap are nevertheless highly useful in the development of therapies^5^,^13^.

Several publications have described strategies for developing and evaluating cancer biomarkers, focusing mainly on biospecimen-derived biomarkers — that is, those derived from patient tissue or biofluids^4^,^16^–^21^. The processes of initial discovery, validation and qualification share many similarities for IBs and biospecimen-derived biomarkers; however, substantial inherent differences exist between both biomarker types (Supplementary information S2 (table)). The FDA and NIH have recognized this distinction and have outlined specific recommendations for image acquisition and analysis in IB development^22^,^23^. Questions of how IB acquisition and analysis should be standardized, and how terminology should be harmonized have been addressed by numerous academic, clinical, industrial and regulatory groups. These groups include the FDA^2^,^24^, the US National Cancer Institute (NCI) through the Quantitative Imaging Network (QIN)^25^ and the Cancer Imaging Program phase I and II Imaging trials initiative^26^, the Quantitative Imaging Biomarkers Alliance (QIBA)^27^,^28^, the American College of Radiology Imaging Network (ACRIN)^29^, the European Society of Radiology (ESR)^30^, the European Organisation for Research and Treatment of Cancer (EORTC) through the QuIC-ConCePT consortium^13^,^31^, the European Association of Nuclear Medicine (EANM)^32^, the International Society for Magnetic Resonance in Medicine (ISMRM)^33^ and Cancer Research UK (CRUK)^34^. Their efforts have produced consensus guidelines for the acquisition and analysis of several IBs^33^–^37^. Many of these organizations also have highlighted the need for a detailed validation and qualification roadmap to improve IB translation^38^,^39^. Recognizing this need, representatives from CRUK and the EORTC, together with other assembled experts in radiology, cancer imaging sciences, oncology, biomarker development, biostatistics and health economics, have formulated an Imaging Biomarker Roadmap for Cancer Studies (FIG. 2). In this Consensus Statement, we outline this roadmap and identify specific considerations for IB validation and qualification, providing illustrative examples of IBs in various stages of development. From this framework, we provide 14 recommendations to accelerate the successful clinical translation of effective IBs, as well as the devalidation^40^,^41^ of IBs that lack utility.

## Current uses of imaging biomarkers

An IB is a measurement derived from one or more medical images. Many IBs are used routinely in healthcare (TABLE 1). IBs provide readily available, cost-effective, non-invasive tools for screening, detecting tumours and serial monitoring of patients, including assessments of response to therapy and identification of therapeutic complications. IBs can enable tracking of a particular tumour repeatedly over time, can map the spatial heterogeneity within tumours, and can evaluate multiple different lesions independently within an individual.

Applications for IBs include the American College of Radiology Breast Imaging-Reporting and Data System (ACR BI-RADS) mammographic breast morphology^42^; clinical tumour, node, metastasis (TNM) stage^43^ (BOX 1); objective response as defined in RECIST version 1.0 (REF. 44) or 1.1 (REF. 45) criteria (BOX 2); bone mineral density T-score measured by dual energy X-ray absorptiometry^46^; and left ventricular ejection fraction^47^ (LVEF; BOX 3). These IBs underpin patient care worldwide, although they are subject to continuing research to improve their performance. In addition, IBs could achieve companion diagnostic status, as seen with the IB [^99m^Tc]-etarfolatide folate receptor positivity^48^ (FR+; BOX 4), demonstrating that imaging can be necessary to guide the choice of therapy for individual patients.

IBs frequently add value in cancer research (TABLE 1). The use of IBs can enable the measurement of patient response to treatment before a survival benefit is observed, which can subsequently lead to early regulatory approval of new drugs^49^ (BOX 2). IBs can indicate the presence of drug targets and target inhibition, for example by proving receptor occupancy^50^ (Supplementary information S3 (box)). IBs have the unique potential to provide serial non-invasive mapping of tumour status during treatment. For example, absolute values of ^18^F-FDG-PET maximum standardized uptake value (SUV_max_) at baseline, or changes in this value observed early during treatment have been used for proof-of-mechanism and proof-of-principle^51^ in drug development (BOX 5), or to demonstrate nonspecific responses to treatment^52^ (BOX 5). Dynamic contrast-enhanced (DCE) CT or MRI-derived *K*^trans^ (REF. 53) (BOX 6) and dynamic contrast-enhanced ultrasonography area under the curve (AUC) values (Supplementary information S4 (box)) have been used as IBs of pharmacodynamic (PD) changes and response to treatment^54^. The use of IBs has led to improved margins of radiotherapy dose delivery^55^ (BOX 5) and surgical margins^56^.

Many more IBs used to measure tumour anatomy, morphology, pathophysiology, metabolism or molecular profiles are being developed in order to study the hallmarks of cancer^57^. Between 2004–2014, approximately 10,000 studies reported new or established IBs (Supplementary information S5 (box)), including IBs derived from new modalities (such as photoacoustic imaging^58^) and new techniques (such as MRI dynamic nuclear polarization^59^; Supplementary information S6 (box)), and new analytical approaches (such as radiomic profiling of tumours to extract multiple features^60^,^61^; Supplementary information S7 (box)), contributing to this ever-increasing number of IBs.

IBs have four key attributes. First, they are a subset of all biomarkers. Second, they can be quantitative or qualitative. Quantitative IBs (measured on an interval or ratio scale^28^) are used in patient care, but other measurements that fall outside this definition (for example, the ACR BI-RADS category, clinical TNM stage, or objective response) are categorical measurements and are also important IBs. In this Consensus Statement, we have deliberately included all image measurements (quantitative or categorical) that satisfy the definition of an IB, according to the current FDA–NIH Biomarker Working Group definition^2^. Third, IBs are derived from imaging modalities, techniques or signals, but are distinct entities; for example, the change in median apparent diffusion coefficient (ADC) is a biomarker, and is distinct from the modality (MRI), the technique (diffusion-weighted imaging) or the measured signal (free induction decay) required to generate the IB (Supplementary information S8 (figure)). Fourth, one imaging measurement can support multiple distinct IBs. For example, ^18^F-FDG-PET has improved disease staging in patients with non-small-cell lung cancer, lymphoma or melanoma by facilitating the identification of nodal and distant metastases^62^. In this case, the IB is clinical TNM stage, which is defined using CT and PET data rather than using CT data alone (BOX 1). Similarly, systems such as PERCIST (for all solid tumours)^63^ and the Deauville five point system (for lymphoma)^64^ aim to improve the assessment of response criteria by incorporating PET data, but produce an ordered categorical IB — namely objective response (BOX 2). This approach is conceptually different from quantifying absolute values of ^18^F-FDG-PET data to derive putative cut-off points subsequently used to identify patients with poor prognosis^65^ or evidence of specific pathway modulation in a clinical trial of an investigational new drug^66^,^67^ (BOX 5).

Many quantitative image measurements comprise continuous data, which must then be categorized to facilitate clinical decision-making. Clinicians often decide between two or more alternative treatment options for each patient, informed by whether a biomarker value is above or below a cut-off point. For example, cancer therapy-related cardiac dysfunction has been defined as LVEF reduction of ≥10 percentage points to a value below normal (53% for adults; BOX 3). Clear guidelines detailing how scintigraphy or 2D echocardiography should be performed have been established for this IB of toxicity for use in healthcare^47^. In distinction, other healthcare-related IBs (such as clinical TNM stage and objective response) are measured as ordered categorical variables. In this case, the boundary between several categories is defined by cut-off points; alternatively, categories can be combined to create a single cut-off point (for example, to select, continue or stop therapy).

In research applications, data are often interpreted as a continuous rather than used in a categorized way. For example, in early phase trials, continuous variable PD biomarkers (including percentage change in tumour size^68^, ^18^F-FDG-PET SUV_max_ (REF. 52),ultrasonography AUC^69^ and MRI median *K*^trans^ (REF. 53) or ADC^70^ (BOXES 5,6; Supplementary information S6 (box)) can indicate antitumour activity of therapeutic agents used at different doses and time points. These studies have led to the demonstration of proof-of-concept and proof-of-mechanism, the definition of pharmacokinetic (PK)–PD relationships and informed on dose selection for novel therapeutic approaches^53^.

## Specific considerations for IBs

IBs and biospecimen-derived biomarkers differ in several important aspects, limiting the relevance to IBs of previous roadmaps (designed for biospecimen-derived biomarkers^13^,^71^; Supplementary information S2 (table)). The performance of imaging devices of different makes and models installed in different clinical centres can vary considerably. These devices are designed, approved, maintained, and operated to provide images^38^ that diagnostic radiologists, nuclear medicine physicians and other clinicians interpret, often with little need to quantify the data obtained. Innovation is largely driven by competition to improve image quality and the user interface; vendors and purchasers often have only secondary interest in how improvements in image quality will affect quantification or standardization of IBs. The measurement of many IBs requires the administration of tracers or contrast agents (for example, diagnostic drugs, usually investigational or off-label), the development and availability of which are uncertain. Many steps in IB validation require a new prospective clinical trial; whenever investigational tracers or contrast agents are involved, the high burden of regulation can cause significant delays in this process.

Many IBs do not purport, even in principle, to measure any underlying analyte, making traditional approaches to assay validation seen with biospecimen-derived biomarkers problematic. For example, dilution linearity and reagent stability — important performance characteristics for biospecimen-derived biomarker assessment^12^ — cannot be assessed for biomarkers based on analysing medical images, such as texture-based IBs. The measurement validity of such IBs depends substantially on events that occur while the patient is coupled to the imaging device. Thus, the role of central reading laboratories in data processing is minor in comparison with biospecimen-derived biomarkers^4^.

## The imaging biomarker roadmap

Biomarkers cross the two translational gaps by passing through a series of domains (discovery, or domain 1; validation, or domain 2; and qualification with ongoing technical validation, or domain 3) that address different research questions. This process follows two parallel and complementary tracks: in one track, technical (assay) performance is examined by addressing whether the biomarker is measurable precisely and accurately, and whether it is widely available in all geographical territories. In the other track, biological and clinical performances are examined by addressing whether the biomarker can be used to measure a relevant aspect of biology or predict clinical outcome. In reality, no biomarker is perfectly validated. Instead, strategies must be defined to identify, mitigate and quantify the uncertainty, risk and cost associated with any given biomarker in making research or clinical decisions^72^–^74^. The nature of these activities and the sequence in which they are combined constitutes a ‘biomarker roadmap’.

In biospecimen-derived biomarker roadmaps, technical (assay) validation commonly occurs early in order to produce a ‘locked-down biomarker’ — that is, the data acquisition and analysis pathways used to measure the biomarker are fixed. In many cases, this stage is followed rapidly by biological and clinical validation because the locked-down biomarker enables widespread evaluation across multiple sites^4^,^18^. For IBs, several key aspects of technical validation (such as multicentre reproducibility) must be addressed at a relatively late stage, unlike biospecimen-derived biomarkers. Studies might address both technical and biological validation concurrently, but progress down each track might be quite independent. As evidence for validation accumulates, a third track of cost-effectiveness must be considered, because IBs must not only demonstrate an association with health benefits, but also demonstrate ‘value for money’ when compared with the use of clinical information alone or with alternative biofluid-based *in vitro* diagnostics^75^.

### Imaging biomarker discovery — domain 1

Most biospecimen-derived biomarkers are molecular features found in the genome, transcriptome, proteome or metabolome that can be chosen rationally to address unmet needs in cancer medicine. This selection approach has also inspired the rational design of several novel targeted nuclear medicine or optical imaging tracers^76^. Many of the most valuable IBs, however, have had a convoluted genesis through unanticipated discoveries in the physical sciences (chemistry, computing, engineering, mathematics or physics) being matched to unmet medical needs after initial discovery, and then developed further.

### Technical validation — domains 2 and 3

Complete technical validation is achieved when an IB measurement can be performed in any geographical location, whenever needed, and give comparable data. Technical validation does not address whether the IB measures underlying biology, or relates to clinical outcome and/or utility, but it can have a major influence on subsequent attempts at qualification because IBs with limited availability or poor reproducibility cannot sensibly undergo further clinical evaluation in large multicentre trials.

#### Precision

Repeatability and reproducibility are related measures of assay precision. Repeatability refers to measurements performed multiple times in the same subject (*in vitro* and/or *in vivo*) using the same equipment, software and operators over a short timeframe, whereas reproducibility refers to measurements performed using different equipment, different software or operators, or at different sites and times^77^, either in the same or in different subjects.

Different intended applications require different levels of evidence for precision^3^. For example, single-centre repeatability might be sufficient to validate a PD or monitoring IB in an early phase clinical trial restricted to that site^53^. Conversely, screening, diagnostic, prognostic and predictive IBs with putative use in whole populations require evidence of reproducibility across multiple expert and non-expert centres before they can be considered technically valid. Such multicentre validation requires complex and costly studies^13^.

Repeatability analysis should be performed early in IB development. Repeatability estimated from studies using rodents can seem unfavourable because, in some models, tumours can grow considerably within a few hours^78^, but useful data can be gained in slow-growing rodent models. Definitive repeatability analysis is best assessed in studies with humans, and should be performed at each centre that evaluates the IB anew, because reliance on historical or literature values is a source of error. Test performance is known to vary between sites, and is influenced by scanner performance, and organ site studied (for example, precision is better in brain lesions^79^ compared with liver or bowel lesions^80^–^82^). Physiological variations between individuals (depending on factors such as caffeine, nicotine, alcohol or concomitant medication) can also alter IB values^83^.

Multicentre studies involve different research institutions that usually utilize devices supplied by different vendors. These devices are broadly equivalent for clinical radiology purposes, although they often have important hidden differences that affect IB acquisition and analysis (Supplementary information S2 (table)). These factors do not preclude multicentre technical assessment of IB precision, but values might not be as reproducible as those derived in few-centre or single-centre studies^84^ (BOX 6; Supplementary information S4 (box)).

Unfortunately, despite the scientific benefits associated with double baseline studies, the time and financial cost of performing such studies often deters investigators and funders from incorporating repeatability or reproducibility evaluations into study protocols. For example, between 2002–2012, only 12 of 86 phase I/II studies of antivascular agents that incorporated DCE-MRI biomarkers, such as the volume transfer co-efficient *K*^trans^, measured test–retest performance^53^ (BOX 6). Multisite reproducibility studies of IBs are very rare.

#### Bias

Technical bias describes the systematic difference between measurements of a parameter and its real value^85^. Few in-human IB studies report bias because real values might not be possible to ascertain in a clinical setting. For some IBs, bias can be estimated by comparison with reference phantoms, as is the case when validating biomarkers on the basis of CT Hounsfield units^86^, and MRI longitudinal (R_1_) and transverse (R_2_) relaxation rates^87^. Imaging phantoms, however, seldom fully represent technical performance in animals or humans. For other IBs, such as the effective transverse relaxation rate (R_2_*), diffusion anisotropy, or *K*^trans^ (all measurable by MRI), appropriate calibration phantoms are not available or poorly represent living tissue.

#### Availability

IB assessments must be feasible, safe, and well-tolerated in the target population, and must have regulatory and ethical approval for use in humans. Differing regulatory approvals (for example, different gadolinium-based contrast agents have been approved by regulatory agencies in Europe, North America and Japan^13^) and commercial pressures (for example, ferucarbotran iron oxide nanoparticles are no longer commercially available in Europe or North America) can substantially affect availability. With regard to PET, some short half-life tracers (such as ^15^O-H_2_O) are highly informative, but require the presence of on-site cyclotron facilities, making worldwide translation impossible with currently available technologies, for economic reasons. Finally, specialist techniques in all modalities require advanced radiographer and technician support, which might not be available locally.

### Biological/clinical validation — domain 2

The terms ‘biological validation’, ‘clinical validation’ and ‘clinical utility’ describe the stepwise linking of biomarkers to tumour biology, outcome variables and value in guiding decision-making, respectively. Clinical validation requires demonstration that the biomarker merely relates to a clinical variable and, therefore, is less difficult to establish than clinical utility (but also less meaningful)^86^. Clinical utility is achieved when the biomarker leads to net improvement of health outcomes or provides information useful for diagnosis, treatment, management, or prevention of a disease^10^,^88^.

The REMARK guidelines^89^ provide a framework for the assessment of clinical utility and validation. Typically, prospective testing of IBs in clinical populations is required in adequately powered studies with follow-up times of 3–5 years to provide outcome data associated with the IB. For example, in patients with colorectal cancer, parameters such as circumferential resection margin^56^, depth of tumour spread, and extramural vascular invasion^90^, are assessed by MRI before surgery, have been validated as preoperative prognostic and predictive IBs, and are currently used to stratify patients into treatment groups. Similarly, ongoing work is evaluating the role of tumour CT, MRI and PET ‘radiomic signatures’ (REF. 91) (Supplementary information S7 (box)) or ^18^F-FDG SUV_max_ baseline values^65^ as prognostic IBs. In these examples, IB data are derived from routine clinical images, making the process of establishing clinical utility much faster than is typically possible for IBs derived from techniques not yet established in routine healthcare^61^.

IBs that have demonstrated clinical validity can have important roles in drug development. For example, RECIST 1.1-defined objective response is a widely used phase II clinical trial end point that has proved useful for preliminary screening of new therapeutic agents^45^. More-rigorous criteria must be met to validate an IB as a trial-level surrogate end point to completely replace a well-established clinical trial end point. The Prentice criteria^92^ describe idealized conditions for demonstration of surrogacy, which unfortunately are rarely achieved and would typically require extremely large datasets^93^. Approaches for surrogate validation more pragmatic than the Prentice criteria involve the application of meta-analysis methods to a collection of large or moderate-sized datasets that measure both the putative surrogate biomarker and the definitive clinical end point^94^. These meta-analytic approaches often require some degree of IB standardization in order to combine datasets in a meaningful manner. Some IBs have been well-standardized (such as the RECIST 1.1 objective response), whereas others (for example, DCE-MRI-derived *K*^trans^^53^; BOX 6) have not. Large prospective multicentre studies relating IBs to an outcome can only be initiated when exhaustive technical validation has established multicentre IB precision and accuracy. Clinical utility (and sometimes, validation) necessarily happens late in the IB roadmap; therefore, alternative validation strategies must be sought for many novel IBs. Biological validation can be approached by accumulating a platform of evidence linking the IB to meaningful biological features, and response to therapies with well-studied mechanisms of action^71^. These principles — outlined by Bradford Hill^93^,^95^ (Supplementary information S9 (box)) — establish evidence for IB performance on the basis of scientific coherence, specificity, strength of association, effect gradient, temporality and consistency.

If the Bradford Hill criteria are adopted, early preclinical imaging–pathology correlation studies^5^,^96^ provide an important component of the platform of evidence because whole-tumour histopathology is rarely possible in patients. Studies conducted with animals enable clinically relevant IBs to be related to fundamental biological processes that can only be measured with invasive techniques. For example, the relationship between DCE-MRI-derived *K*^trans^ and tumour blood flow rate (a fundamental measure of response to antivascular agents) was investigated using a terminal radiotracer-based technique in rat tumour models^97^. Preclinical studies also enable the examination of the therapeutic dose–response relationship at different time points in a range of tumour models. Of note, however, pathophysiological assays are often considered as a ‘reference standard’, but are also biomarkers and thus imperfect, and also subject to sampling bias. Thus, some ‘reference standard’ tests might not enable the prediction of survival, or relate to the intended biological process. Moreover, precise pathophysiological correlates of some IBs might not exist, or can be almost impossible to obtain^13^.

Evidence of imaging–biology correlations and early response to therapies can provide sufficient biological validation to establish IBs as useful for PD assessments or monitoring response in early phase clinical trials, even in the absence of compelling outcome data^53^. The IBs ^18^F-FDG-PET SUV_max_ and *K*^trans^ (BOXES 5,6) are illustrative of how IBs can cross translational gap 1 to guide decision-making for subsequent studies. For example, the biologically active dose for the antivascular agent ZD6126 calculated according to *K*^trans^ measurements^98^ was shown to be greater than its maximum tolerated dose, effectively halting further clinical development of this agent. In other studies, *K*^trans^ data informed of the biologically active dose for cediranib^99^ and the optimum scheduling for brivanib^100^ (BOX 6).

### Cost-effectiveness — domains 2 and 3

To be translated into the clinic, IBs must provide good ‘value for money’ and compare favourably with biospecimen-derived biomarkers resulting from technologies, such as ‘liquid biopsies’ of isolated circulating tumour cells or cell-free DNA. In the research setting, the value added by testing a key hypothesis (with an IB) should be greater than the cost of performing the study. In healthcare, the economic test is harsher, because even well-validated IBs will not cross translational gap 2 unless they offer an advantage in terms of cost per quality adjusted life year (QALY) gained^13^. Initially, translating IBs into the healthcare setting is costly and time-consuming^101^.

Studies in early stages of IB discovery and validation can access conventional biomedical research funding streams, whereas evidence-gathering studies to support IB qualification can be difficult to fund. For example, large-scale multicentre reproducibility studies can be unattractive to research funders if seen as ‘incremental’ and, unless such studies create exploitable intellectual property, they are not attractive to commercial sponsors. One approach to solve this problem is to assemble consortia of commercial and not-for-profit stakeholders, such as the QuIC-ConCePT consortium^13^,^31^, QIBA^28^ and QIN^102^, to undertake multicentre validation steps to meet the collective needs of the community.

Initial IB translation requires large-scale funding by government, charity or industry, or commercialization of the IB by an imaging company (for example, a major scanner manufacturer in the case of hyperpolarized ^13^C-MRI/MRS), or by a start-up business focused on a specific technique or IB. This strategy requires careful assessment of the likely risk–benefit of the development process, and strong intellectual property positions to ensure likely financial return on the imaging device, agent or biomarker^38^. Complex economic considerations are related to IBs developed as companion diagnostics associated with a specific therapeutic. Such IBs might be cost-effective for the healthcare payer (reducing futile expenditure and avoiding adverse effects in patients who cannot benefit from the drug) as well as beneficial for the supplier of the therapeutic (leading to a reduction in the size of trial cohorts, enabling marketing authorization, and providing better ratios of costs per QALY gained), but are associated with increased risks for the IB developer because, if clinical translation of the therapy fails (such as in the case of vintafolide^48^; BOX 4), the market for the companion diagnostic disappears.

### Qualification — domain 3

The term ‘qualification’ has different scientific and regulatory meanings. Generally, qualification is an evidentiary process of linking a biomarker with biological processes and clinical end points intended to establish that the biomarker is fit for a specific purpose^96^,^103^,^104^. For example, the use of IBs qualified as prognostic would enable the enrichment of a clinical trial population with patients at the highest risk of experiencing a clinical event, thus increasing the efficiency of the trial^105^. Such IBs have to be clinically validated to show their association with an outcome, but might not necessarily be associated with clinical utility for decision-making in healthcare. In distinction, the same IB could potentially be qualified for prediction of patient response (or lack of response) to a particular therapy based on evidence that the treatment effects in biomarker-positive patients differ from those in biomarker-negative patients (for example, superior outcome with experimental therapy in biomarker-positive patients versus biomarker-negative patients)^106^. The IB might be used for enrichment or stratification of patient populations in clinical trials and, if the trial is successful, such IB could be further developed into a companion diagnostic with established clinical utility for identifying patients likely to benefit from the new therapy.

Qualification requires demonstration of fitness for a particular purpose and, therefore, an IB might need to be evaluated in several scenarios to justify a broad claim of qualification. Hence, the IB might become qualified for one particular use (such as for a cancer type or a certain drug class), but not for others. Nevertheless, when a biomarker is qualified in multiple settings (for example, different tumour types, different therapies or different research questions), then the process of qualification for a new application can be expedited because most of the necessary validation requirements are likely to have been fulfilled^107^.

‘Regulatory qualification’ is a more-specific term that describes a framework for the evaluation and acceptance of a biomarker for specific use in regulatory decision-making^108^ (Supplementary information S1 (table)). Examples include new drug approvals or safety monitoring. This framework for regulatory qualification is not a formal requirement for a drug developer who merely wishes to ‘qualify’ an IB to support a proof-of-mechanism or a dose-selection decision.

## Roadmap application

**Figure 2:**
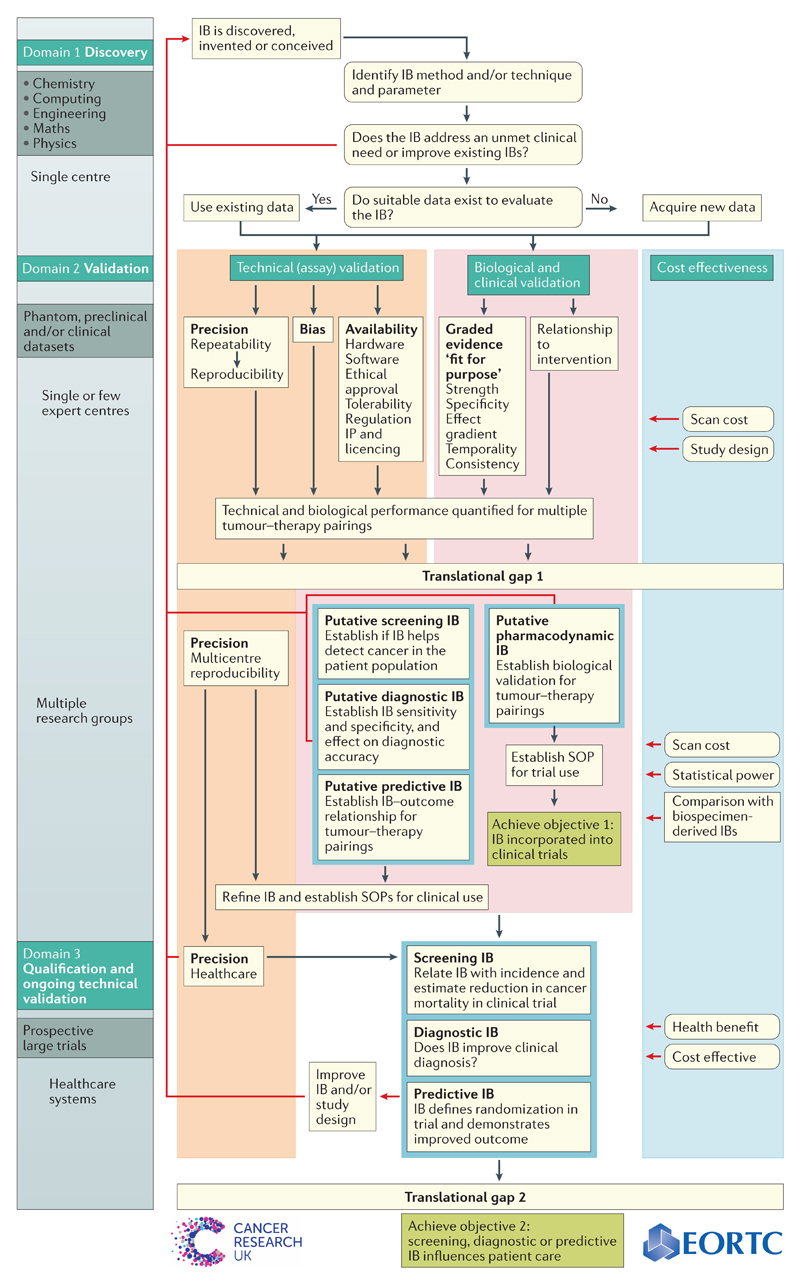
The imaging biomarker roadmap. A detailed schematic roadmap is depicted. The imaging biomarker (IB) roadmap differs from those described for biospecimen-derived biomarkers. For imaging, the technical and biological/clinical validation occur in parallel rather than sequentially. Of note, essential technical validation occurs late in the roadmap in many cases (such as full multicentre and multivendor reproducibility). Definitive clinical validation studies (IB measured against outcome) are deferred until technical validation is adequate for large trials. In the absence of definitive outcome studies, early biological validation can rely on a platform of very diverse graded evidence linking the IB to the underlying pathophysiology. Cost-effectiveness impacts on the roadmap at every stage, owing to the equipment and personnel costs of performing imaging studies. Technical validation and cost-effectiveness are important for IBs after crossing the translational gaps because hardware and software updates occur frequently. Therefore, technical performance and economic viability must be re-evaluated continuously. SOP, standard operating procedure. Image reproduced from http://www.cancerresearchuk.org/sites/default/files/imaging_biomarker_roadmap_for_cancer_studies.pdf.

With this Consensus Statement we aim to accelerate IB translation. For this purpose, we have produced 14 key recommendations (BOX 7), accompanied by a detailed imaging biomarker roadmap for cancer studies (FIG. 2). Together, the roadmap and recommendations provide a framework for understanding how to examine qualitative and quantitative IBs at all stages of their validation and qualification. Herein, we identify the key steps required to achieve these goals, recognizing the important differences between IBs and biospecimen-derived biomarkers. No biomarker is expected to be perfect; instead, this roadmap provides a tool to assess the current evidence for technical and biological and/or clinical performance of any given IB at any stage of development. The limitations of each IB can be identified, quantified, documented and made publicly available. In the process of creating this roadmap, we have identified several potential obstacles in the clinical translation of putative IBs.

### Key recommendations

#### Recommendations 1–2 — grant submissions and study publications

Proposals for funding to support IB-related studies should state clearly how these will advance IB validation or qualification. Resulting journal publications should state explicitly how these aims have been achieved (recommendation 1). Study design, protocols, quality assurance processes and standard operating procedures should be reported exhaustively by making full use of supplementary materials for research publications. The software used for image processing should also be reported and be made available once intellectual property rights have been addressed (recommendation 2). These two recommendations will result in the greatest possible confidence in the reported IB-related data, will facilitate the conduct of statistically valid meta-analysis studies of imaging data, and will help investigators, reviewers, target audiences, funders and governments evaluate the risks associated with each IB for any given research or healthcare application.

#### Recommendations 3–7 — technical (assay) validation

A compelling rationale supports the accreditation of clinical imaging laboratories as being competent for measuring a given IB (recommendation 3), in line with the standards set by the biospecimen-derived biomarker community^4^. This approach has been adopted by the PET community when performing quantitative ^18^F-FDG-PET in clinical trials both in Europe and North America, focusing on performing regular equipment calibration and quality assurance. Moreover, data acquisition, analysis and reporting standards have been adopted^32^. In Europe, this initiative has been driven by the EANM and endorsed by the EORTC to ensure that institutions involved in multicentre clinical trials adopt best-practice procedures, and that quantitative reporting is harmonized across sites to improve reproducibility. Participants receive certification to distinguish them from nonparticipating institutions. Similarly, standardization of DCE-ultrasonography^109^ and LVEF measurements^47^ have been addressed by expert consensus. In the USA, the NCI evaluates performance of dynamic and static PET, volumetric CT and DCE-MRI in partnership with the ACRIN^29^. All NCI-designated cancer centres have been certified for measurement value equivalence, with ongoing annual inspection to provide regulation^39^. This certification involves the incorporation of IBs in clinical trial design and requires evaluation of the performance of imaging sites by the NCI Clinical Trials Network^26^.

Site accreditation is an important step towards improving technical performance in multicentre studies, but must reflect widely sought academic consensus, become adopted by international societies, and receive the backing of funders, industry and regulators for such accreditation to have value. Accreditation must simultaneously promote standardization and harmonization of IBs for multicentre use, while accommodating studies led by investigators who have scientific freedom to further develop and optimize IBs. To achieve this optimization, best-practice guidelines for each widely used IB (or related family of IBs) must be updated and reviewed regularly (recommendation 4), because biomarker drift is inevitable owing to technological advances in scanner performance (for example, clinical trials with ongoing data collection can be affected by hardware and software upgrades). Suitable statistical methods, such as multivariate linear regression analysis and other more complex statistical approaches, must be used to adjust for changes in IBs during ongoing studies (for example, defining pre-change and post-change data).

IB precision must be demonstrated early in IB development through single-centre repeatability studies, or fewsite reproducibility studies (recommendation 5). This assessment is particularly important when testing the ability of an IB to measure the effect of therapeutic intervention. The choice of performance metric is important; for example, the coefficient of variation assumes that the standard deviation is approximately proportional to the mean. If studies of repeatability are performed under multiple conditions (such as imaging patients across different groups), then a plot of the standard deviation versus mean should be examined to determine whether this proportionality is valid. In most imaging studies of repeatability, only two replicates are acquired for each patient and thus, a Bland Altman plot will enable the evaluation of whether or not the mean of the measurements influence on variance, providing overall limits of agreement^110^.

Once the IB is shown to have sufficient technical and biological validity to cross translational gap 2, multicentre reproducibility must be evaluated (recommendation 6). Some studies have measured IB multicentre reproducibility (for example, DCE-CT evaluation of ovarian cancer^111^), but these are rare exceptions. The recruitment of patients with cancer to attend for scanning on devices at multiple centres is seldom possible and thus, studies usually require the use of data from different patients, scanned on different machines. Considerable centre-specific differences might exist regarding devices, contrast agents and tracers, and software. This variability must be accounted for when considering multicentre reproducibility. Mixed-effects modelling provides a statistically robust approach to maximising data inclusion, while acknowledging inevitable slight inconsistencies in the data^112^.

Data-analysis strategies must be developed for multicentre studies (recommendation 7). Analysis led by one central site can reduce data variation for studies with a moderate number of participating sites^113^,^114^. As IBs transition towards crossing gap 2, however, using one central site is inappropriate for IBs that will eventually be analysed at many cancer centres once the IB has been adopted in healthcare. To facilitate this transition, sites should compare their own technical performance against a central analysis, similarly to the assessment of objective responses in oncology trials.

#### Recommendations 8–11 — biological validation and clinical validation

Clinical validation occurs relatively late in the development process for most IBs compared with biospecimen-derived biomarkers^71^. Extensive preclinical studies can provide well-controlled data to examine the relationship of the IB to pathology, and the IB to the effects of interventions, and therefore are strongly encouraged (recommendation 8). The choice of experimental model is an important consideration. Tumour xenograft models in immunodeficient mice are well-studied and have reproducible growth characteristics^115^, and can be ideal for initial IB development, but tumour models that better portray the relevant biological characteristics found in human cancers are also needed^116^,^117^. Firstly, *in situ* tumours, or those implanted into their orthotopic site often better recapitulate the local microenvironment of human tumours^117^,^118^. Secondly, syngeneic models, with intact immune systems, are essential for some studies (for example, for the evaluation of IBs for immunotherapies). Thirdly, appropriately genetically-engineered mouse models can have lesions that accurately mimic human tumours^119^, an approach that can facilitate co-clinical trials in which preclinical studies are run in parallel with clinical trials in order to identify likely responders to targeted therapies^118^,^120^. Finally, models derived from patient tissue (PDX models)^121^ or circulating tumour cells (CDX models)^122^ offer potential insights into developing personalized therapeutic regimens^123^. The biological validation of IBs must incorporate the use of these models once proof-of-concept has been demonstrated in xenograft models. When possible, IBs should then be validated in clinical studies^124^, in order to confirm imaging–biology relationships in humans. Adaptive trial designs can be useful for early stage IB studies^125^. Sample-size re-estimation can be performed in those studies in which limited relevant data inform on sample size^126^. Similarly, group-sequential design^127^ can provide flexibility in the number of animals or patients entered into a study. Such approaches can ensure adequate power with small sample sizes — if effect sizes are suitably large — and, therefore, can make imaging studies more affordable^128^.

Improved methods for imaging–biology correlation are needed for IB biological validation (recommendation 9), because currently available methods often fail to account for extensive spatial heterogeneity in the IB^129^ and in the tissue pathology^118^, and for the difference in scale of the two measurements. Moderate-level concordance between imaging data (slice or volume) and pathology (single section) provide only limited biological validation. Instead, 3D comparisons are strongly encouraged, to better co-localise imaging and pathology data and reduce sampling bias. In some studies, genomic, transcriptomic and proteomic biomarkers, or biofluid-derived biomarkers, including circulating tumour cells, might be more-appropriate validation tools than tissue-derived measurements^91^,^130^. The use of appropriate statistical methods is required to define associations between imaging and pathology measurements. Spearman’s *rho* or Kendall’s *tau* rank correlation coefficients enable the comparison of IB values to reference standard pathology readouts within a cohort; however, if these two measurements (or transformations of these variables, such as logarithms) relate linearly to one another, regression models provide estimates of biases and the scale of the relationship.

IB studies often generate rich datasets that can be collected and banked for future re-use. These data include raw images, processed images and parameter maps, ancillary files (such as regions of interest, mask files, and stored header information detailing scan parameter settings), and essential patient metadata covering demographics, treatment history and clinical outcome (such as response, progression-free survival and/or overall survival). Useful data-archiving systems require financial support; collaboration between multiple academic, industry and funding partners; and ongoing curation and active management — as exemplified by the NCI Informatics Technology for Cancer Research platform^131^. Technological advances (such as cloud-based solutions) must be accompanied by suitable information governance arrangements, potentially crossing international legal and regulatory frameworks^102^, and by commitment from funders to resource these initiatives.

Creation of animal and human cancer image repositories, following the lead of organizations including the NCI and the ESR^132^, is strongly encouraged (recommendation 10) to enable rapid testing of new analyses by researchers from different institutions. In some cases, this approach can even lead to a reduction in the number of animal experiments (in line with the 3Rs of animal welfare in cancer research^116^) or in the number of new patients recruited. Standardized data collection, analysis and archiving are required to support multisite clinical trials^102^.

Complete and transparent study reporting is essential to avoid selective reporting bias and publication bias (recommendation 11). Results of all prespecified analyses (secondary analyses as well as primary analyses) should be reported, regardless of whether they are consistent with expectations, or whether they achieve statistical significance^133^,^134^. Highlighted exploratory analyses (either prespecified or *post hoc*) should be accompanied by a description of all other exploratory analyses performed. This strategy will more-accurately reflect the potential for false-positive and false-negative findings. Adherence to these principles of reporting will help to eliminate the distortion of research findings resulting from selective reporting and failure to publish negative studies. These biases can also be reduced by registering studies and detailing their hypotheses on publically available websites (such as ClinicalTrials.gov) before the study is initiated or before outcome data are unblinded^89^,^135^.

#### Recommendation 12 — qualification

Robust study design and predefined statistical analysis plans are vital to ensuring that the highest quality evidence is available to qualify IBs. Late-stage multicentre clinical trials should be powered adequately to demonstrate clinically useful effects. At present, few IBs are rigorously validated or qualified as prognostic for quality of life, progression-free survival or overall survival^136^. Appropriate statistical methods (for example, control of the false-discovery rate and cross-validation techniques^137^) are needed to avoid spurious findings and overfitting caused by measurement of large numbers of image parameters relative to numbers of patients — a common problem in several published biospecimen-based biomarker studies^138^. Efficient study designs should be pursued, for example, in studies of diagnostic accuracy^135^ or test impact, in which each patient is regarded as his or her own control; examples include adding functional imaging to standard anatomical radiology in order to improve diagnosis^139^,^140^. In later-stage clinical trials, several treatments can be tested concurrently in the same trial using enrichment or predictive IBs, as is the case of current molecular profiling-based studies^141^. This measure can increase recruitment efficiency, reduce costs, and accelerate achievement of primary end points^142^.

Qualification requires rigorous and detailed statistical reporting standards. Screening and diagnostic accuracy IB studies should report sensitivity and specificity, results of receiver operating characteristic (ROC) curve analysis, and negative and/or positive predictive values^135^, whereas prognostic and predictive IB studies should report estimated effect sizes, such as hazard ratios89. Estimates should be accompanied by measures of uncertainty (for example, 95% confidence intervals) as well as statistical significance. In studies in which predictive biomarkers are evaluated using randomized controlled trials, CONSORT guidelines should be followed^143^.

#### Recommendations 13–14 — cost-effectiveness

New models for funding and regulation should be developed for investigational devices, tracers and contrast agents, and software that have value as IBs in the research setting, but lack commercial viability as a diagnostic product in healthcare systems (recommendation 13). Imaging studies are perceived to be expensive and thus, integration of investigational IBs should be linked to existing radiological tests (addition of the IB to a clinically approved ultrasonography, CT or MRI examination, for example) whenever possible. Large-scale studies should include health-economic considerations, including measuring the cost-effectiveness of IBs versus competitor tests (other IBs or biospecimen-derived biomarkers)^144^ (recommendation 14).

## Conclusions

Clinical imaging has transformed contemporary medicine^145^. IBs have enormous potential to facilitate further advances in cancer research and oncology practice by accurately informing clinical decision-making, but must undergo rigorous scrutiny through validation and qualification to achieve this end^13^. This process of evaluation enables investigators and consumers to make informed decisions about IB translation for each research and healthcare application^10^. The roadmap and recommendations that we present herein will, if adopted, mark a change in the development and use of IBs in cancer research and patient management.

## Supplementary Information

**See online article:** S1 (table) | S2 (table) | S3 (box) | S4 (box) | S5 (box) | S6 (box) | S7 (box) | S8 (figure) | S9 (box)

**All Links are Active in the Online Pdf**

Supplementary information S1

Supplementary information S2

Supplementary information S3

Supplementary information S4

Supplementary information S5

Supplementary information S6

Supplementary information S7

Supplementary information S8

Supplementary information S9

## Figures and Tables

**Figure 1:**
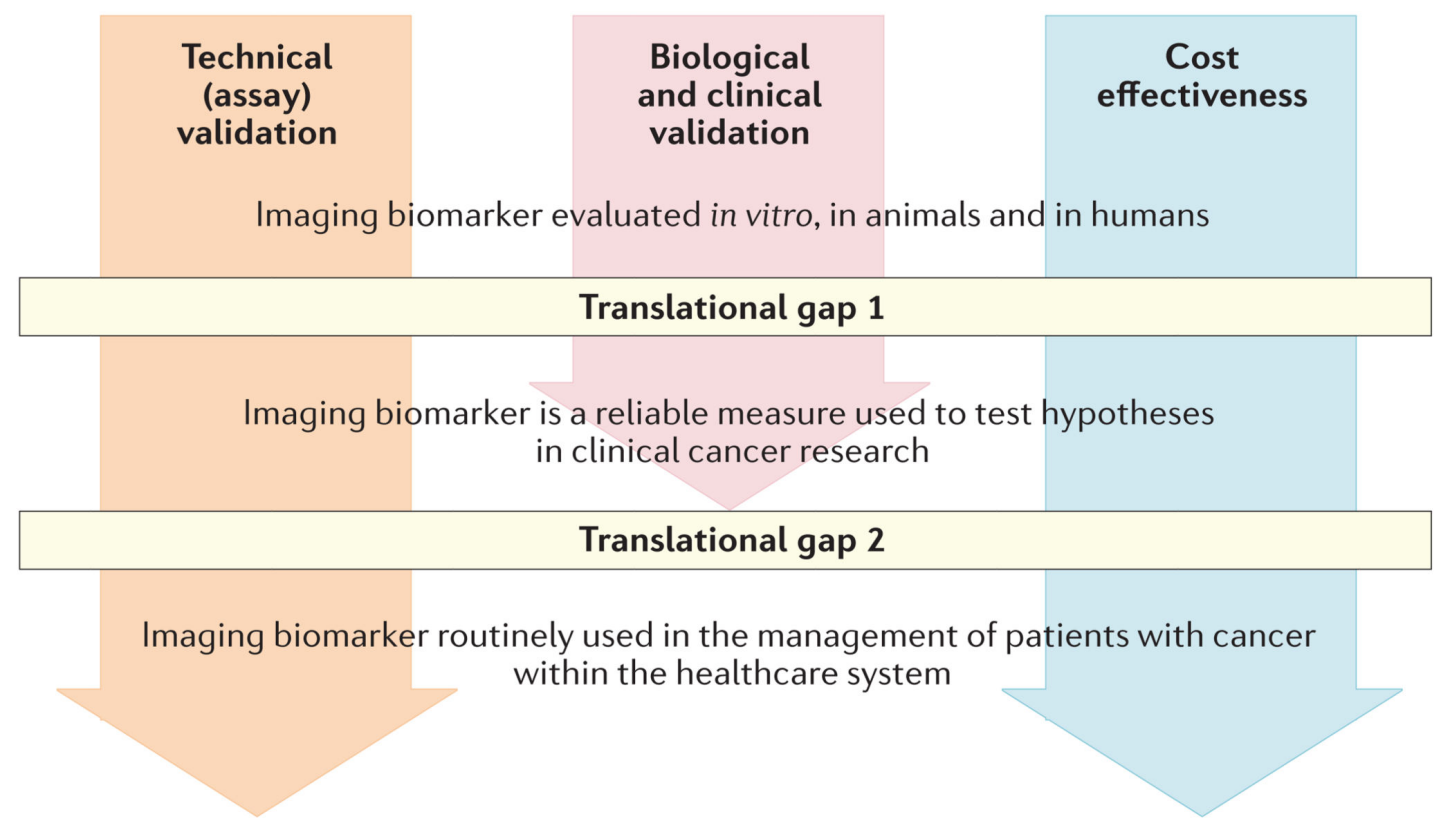
Overview of the imaging biomarker roadmap. Imaging biomarkers must cross translational gap 1 to become robust medical research tools, and translational gap 2 to be integrated into routine patient care. This goal is achieved through three parallel tracks of technical (assay) validation, biological and clinical validation, and cost effectiveness.

**Table 1:** Selected list of imaging biomarkers used in clinical oncology decision-making

Biomarker	Modality	Decision-making role	Notes	Refs
***IBs that have crossed translational gap 2 into healthcare***				
ACR BI-RADS breast morphology	Mammography	Diagnostic in breast cancer	Used worldwide	42
Clinical TNM stage	XR, CT, MRI, PET, SPECT, US, endoscopy	Prognostic in nearly all cancers	Used worldwide Guides management of nearly every patient with a solid tumour Extensively validated and qualified	43
Bone scan index	SPECT	Prognostic in prostate cancer	Continuous variable data converted to ordered categorical IB Calculation uses software requiring regulatory approval	164, 165
Left ventricular ejection fraction	Scintigraphy, US	Safety biomarker Guides therapy	Guides management of a substantial number of patients (for example, trastuzumab) Decrease in LVEF of >10% confirmed with repeated imaging	150
T-score	DXA	Safety biomarker Guides prescription of bisphosphonates to patients with breast cancer and bone loss induced by therapy	Number of standard deviations below mean bone density Calculation uses software requiring regulatory approval	46
Uptake of ^111^In-pentetreotide, ^68^Ga-dotatate octreotide conjugates	SPECT, PET	Identification of primary or residual neuroendocrine lesions Prescription of ^177^Lu-dotatate-octreotide ablation therapy	IB is SUV_max_ (target lesion) >SUV_max_ (background liver or bone marrow)	152, 153
^99m^Tc-tilmanocept uptake above cut-off	SPECT	Intraoperative detection of sentinel lymph nodes	Biomarker cut-off is background radioactivity counts >3 standard deviations from the mean background count level, with background counts determined from tissue at least 200 mm distal to the injection site Approved for use in patients with breast cancer or melanoma	166
Split renal function measured by ^99m^Tc-mertiatide (MAG3)	SPECT	Determination of split renal function prior to nephrectomy, which guides surgical decision-making	NA	NA
MARIBS category	MRI	Determination of risk of breast cancer in patients harbouring genetic risk factors such as mutations in *BRCA1* or *BRCA2*	Approved by NICE for clinical use in UK	154
Objective response	CT, MRI, PET	Guides decision to continue, discontinue, or switch therapy	Used worldwide to guide management of nearly every patient with a solid tumour Extensively validated and qualified	44
Circumferential resection margin status	MRI	Determination of whether circumferential resection margin is clear in rectal cancer with pre-operative high-resolution MRI scan	Prognostic value in rectal cancer; now approved for clinical use	56
***IBs approved by FDA as surrogate end points***				
Objective response	CT, MRI, PET	End point in phase II trials Contribution to PFS determination	PFS end point is heavily based on objective response as well as serology and clinical markers	49
Splenic volume	CT, MRI	Assessments of response in patients with myelofibrosis	Used in FDA approval of ruxolitinib	49
***IBs evaluated by EMA as companion diagnostics***				
^99m^Tc-etarfolatide FR+	SPECT	Assessment of FR+ status with ^99m^Tc-etarfolatide recommended by CHMP as a companion imaging diagnostic in patients with platinum-resistant ovarian cancer receiving vintafolide	Recommendation conditional on the outcome of the phase III PROCEED trial, which unfortunately had negative results	155, 167
***IBs that have crossed translational gap 1 into therapeutic trials and hypothesis-driven medical research***				
Left ventricular ejection fraction	Scintigraphy, US	Safety biomarker Guides decision to stop therapy	Guides recruitment and continuation in many clinical trials Decrease in LVEF >10% confirmed with repeated imaging	47
AUC	US	Pharmacodynamic and putative predictive IB	Reduction in DCE-US AUC at 1 month following antiangiogenic therapy has been shown to predict freedom from disease progression and overall survival	168
^18^F- FDG SUV_max_	PET	Used for regional selective dose boost	Ongoing clinical trial	55
Δ^18^F- FDG SUV_max_	PET	Pharmacodynamic biomarker in pharmacological audit trail Monitoring IB for other therapies Used in dose-finding and to provide evidence of efficacy	Change in ^18^F-FDG-PET SUV_max_ is becoming a useful IB in single-centre studies of drugs that inhibit the PI3K-AKT-mTOR pathway	67
Δ*K*^trans^ (and related IBs)	CT, MRI	Proof-of-concept Used for dose-finding Informs go/no-go decision-making on the basis of biologically active dose versus MTD Used for dose-scheduling	Change in *K*^trans^ is a consistently useful IB in single-centre studies of drugs that target the tumour vasculature Baseline *K*^trans^ has consistently failed to demonstrate value as an outcome IB	53
Receptor occupancy (%)	PET	Pharmacological audit trail evidence of target engagement	Receptor occupancy measured for the neurokinin-1 receptor antagonist aprepitant	50

ACR BI-RADS, American College of Radiology Breast Imaging-Reporting and Data System; AKT, RAC-alpha serine/threonine-protein kinase; AUC, area under the curve; BRCA1/2, breast cancer type 1/2 susceptibility protein; CHMP, Committee for Medicinal Products for Human Use; DCE, dynamic contrast-enhanced; DXA, dual-energy X-ray absorptiometry; EMA, European Medicines Agency; FR+, folate receptor-positive; IB, imaging biomarker; *K*^trans^, volume transfer coefficient; LVEF, left ventricular ejection fraction; MARIBS, magnetic resonance imaging in breast screening; MTD, maximum tolerated dose; mTOR, mechanistic target of rapamycin; NA, not applicable; NICE, National Institute for Health and Care Excellence; PI3K, phosphoinositide 3-kinase; SPECT, single-photon emission computed tomography; SUV_max_, maximum standardized uptake value; US, ultrasound; XRT, X-ray computer tomography.

## Box 1 Clinical TNM stage – prognostic and predictive IB

Staging systems record the presence, size and number of lesions at tumour, nodal and other metastatic sites to derive an ordered categorical biomarker of patient disease burden. The American Joint Committee on Cancer (AJCC) Cancer Staging Manual^43^ provides detailed radiology reporting guidelines, enabling measurements to be robustly reproduced. For solid tumours, tumour, node, metastasis (TNM) staging systems or equivalents derived from imaging modalities, including CT, MRI, single-photon emission computed tomography (SPECT; for example, ^99m^Tc-bone scintigraphy) and PET (for example, ^18^F-FDG-PET), either alone, or supported by other biospecimen or clinical measurements are available (see figure below). TNM stage is prognostic in nearly all cancer types.

TNM is also predictive in some settings. In prostate cancer, clinical TNM stage distinguishes localized disease (T1–2 N0/Nx M0) from locally advanced disease (T3–4 any N M0; or any T N+ M0). This imaging biomarker (IB) is prognostic because patients in the former group have worse outcomes than those in the latter group. The same IB, however, is predictive of benefit for bicalutamide monotherapy, because this treatment only benefited patients with locally advanced disease in a large randomized controlled trial, leading to approval of the drug in the UK^146^. This IB has crossed the two translational gaps in biomarker development and is used daily in clinical practice.

## Box 2 Objective response – monitoring and response IB

Response criteria (such as complete response, partial response, stable disease or progressive disease) define the patient’s response to therapy as an ordered categorical measurement. Solid tumours are assessed by WHO^147^, RECIST 1.0^44^ and 1.1^45^ or similar criteria using imaging techniques alongside biospecimen-derived and clinical measurements (see figure below). Response criteria require a great level of detail and therefore, such measurements can be reproduced robustly.

Objective response has crossed the two translational gaps in biomarker development and is used daily in clinical practice, as well as by regulators to approve new drugs for full market approval and accelerated approval^49^. Several research studies have attempted to optimize the definition of objective response for specific tumour–therapy combinations. Examples include incorporating ^18^F-FDG-PET data into response assessment in lymphoma, measuring the peak of the standardized uptake value (SUV_peak_) in PERCIST^62^ and the peak of the standardized uptake value (SUV_max_) in the Deauville criteria^63^, or adapting thresholds required to define partial response and partial disease as described in the Choi criteria for assessing response of GIST to imatinib therapy^148^. In all of these cases, however, the biomarker remains the objective response.

When a new version of a biomarker is tested against an existing version (for example, PERCIST and RECIST 1.1 definitions of objective response), and if the possibility of comparing paired measurements from each patient (with established and fixed acquisition and analysis methods) exists, then assessment on whether the new method better predicts a relevant clinical end point (such as overall survival) can be carried out. However, information can also be derived from measuring concordance between the two methods on a per-case basis (with a weighted *kappa* statistic) because both methods might measure different aspects of tumour biology or have different error sources, but can have similar ability to predict a clinical end point.

## Box 3 Left ventricular ejection fraction – safety prognosis and monitoring IB

Many well-established (for example, radiotherapy, doxorubicin or trastuzumab) and recently introduced (for example, tyrosine kinase inhibitors) anticancer therapies are associated with a substantial risk of cardiotoxicity^149^. Reduced left ventricular ejection fraction (LVEF) is well-established in cardiovascular medicine as prognostic of cardiac outcomes, measured by various methods, such as transthoracic ultrasonography, scintigraphy and, in research studies, using MRI. Guidelines for the use of LVEF in the care of patients with cancer have been defined, with a consensus in defining cancer-therapeutics-related cardiac dysfunction as a decrease in LVEF of >10%, confirmed with repeated imaging^47^.

This imaging biomarker (IB) has crossed the two translational gaps in biomarker development and is used widely in care of cancer patients; FDA-approved labels for therapies such as lapatinib, sunitinib, doxorubicin or trastuzumab^150^ all require measurement of LVEF.

## Box 4 ^99m^Tc-etarfolatide — companion diagnostic IB with regulatory approval

Nuclear medicine imaging biomarkers (IBs) can help to identify the presence or absence of a cancer phenotype, acting as binary categorical biomarkers, similarly to genomic identification of mutation status. For decades, ^131^I-radio-iodine scintigraphy has enabled the identification of local and distant disease in patients with thyroid cancer, as a subjective radiological assessment, rather than by deriving an IB^151^. Conversely, radiolabelled somatostatin receptor analogues are used in scintigraphy (for example, ^111^In-pentetreotide octreotide)^152^ and in PET–CT (for example, ^68^Ga-dotatate)^153^ to identify neuroendocrine tumour sites; such IBs are categorical, defined by a maximum standardized uptake value (SUV_max_) in the target lesion that exceeds the background SUV_max_. If the scan is positive, the IB alters clinical management because ablative treatment is given (see figure below).

The European Medicines Agency (EMA) and the FDA have authorized new personalized medicines with a label mandating a blood-based, tissue-based, or imaging-based companion diagnostic. As an example of how IBs could be companion diagnostics in oncology, the EMA Committee for Medicinal Products for Human Use recommended FR+ (folate receptor-positive status assessed using ^99m^Tc-etarfolatide scintigraphy) for approval as a companion imaging diagnostic in patients with platinum-resistant ovarian cancer treated with vintafolide therapy. This recommendation was conditional on the outcome of the PROCEED trial of vintafolide, which reported negative results^154^,^155^. This case set an important regulatory precedent for an imaging-based companion diagnostic.

## Box 5 ^18^F-FDG-PET SUV_max_ — a monitoring, PD and radiotherapy planning IB

This imaging biomarker (IB) is distinct from the contribution of ^18^F-FDG-PET signals to define clinical TNM stage or objective response. ^18^F-FDG-PET SUV_max_ (maximum standardized uptake value) has crossed translational gap 1 in three distinct settings:

- ^18^F-FDG-PET signals map the spatial variation in glucose uptake, which is increased in tumours owing to their high rate of glycolysis^32^,^35^. Many therapies reduce glucose uptake, including established cytotoxic chemotherapy agents^156^ and antiangiogenic agents^66^,^157^; for such therapies, Δ^18^F-FDG-PET SUV_max_ is a nonspecific IB for monitoring treatment response.
- Conversely, over 20 studies of PI3K-AKT-mTOR pathway inhibitors have used Δ^18^F-FDG-PET SUV_max_ as a pharmacodynamic (PD) IB to measure specific and very acute effects of target and pathway inhibition^67^,^158^,^159^. In these studies, Δ^18^F-FDG-PET SUV_max_ provides proof-of-mechanism in the pharmacological audit trail^5^ as a specific PD IB, because PI3K plays a central role in regulating cell functions, including metabolism, in conjunction with downstream kinases, such as AKT and mTOR^160^ (see opposite image).
- The spatial distribution of ^18^F-FDG-PET SUV_max_ can be mapped and used as an IB in radiotherapy treatment-planning. A randomized phase II trial is comparing isotoxic dose-escalation and dose-boost to tumour regions with >50% of the SUV_max_ value^55^. If the results of this study are positive, it could support the use of functional imaging in radiotherapy planning and adaptive therapy during treatment.

Further standardization is necessary for the IB to cross translational gap 2.

## Box 6 Δ*K*^trans^ and Δ*IAUC*_60_ — monitoring and PD IB

*K*^trans^ is a composite measurement of blood flow and vessel permeability^34^, derived from MRI or CT data — with both modalities showing reasonable levels of agreement^161^,^162^. Change in *K*^trans^ from baseline (Δ*K*^trans^) and similar metrics (such as Δ*IAUC*_60_ (integrated area under the curve at 60 seconds)) have been used in >100 early phase clinical trials and academic-led studies of antivascular agents to demonstrate proof-of-principle^51^,^82^, as well as to identify optimum drug doses and schedules^53^ (see figure below).

Δ*K*^trans^ has crossed translational gap 1 as part of the pharmacological audit trail^5^, and has assisted in the development of antiangiogenic drugs by helping to select doses for cediranib^99^ and other agents^53^, and determining the schedule for brivanib^100^. This IB has also contributed to the decision to halt further development of the antivascular agent ZD6126 by demonstrating that the biologically active dose was greater than doses that induced toxicity^98^.

At present, substantial interlaboratory variation in DCE–MRI acquisition and analysis mean that absolute values of *K*^trans^ can vary by an order of magnitude across centres^163^. This limited technical validation has prevented IBs based on absolute values of these parameters from crossing the translational gaps to be used as prognostic IBs^136^.

## Box 7 Recommendations for imaging biomarker validation and qualification in cancer studies

### Technical (assay) validation

1. Clinical laboratories and imaging centres should be accredited for IB acquisition and analysis. Academic, clinical, industry and regulatory partners must work across each jurisdiction to develop, maintain and regulate the necessary framework to achieve this accreditation.
  *Rationale.* Good-quality IB studies are difficult to perform. Accreditation is a vital step in biospecimen-derived biomarker validation^4^,^17^ and similar steps are required for IBs.
2. The imaging community (academics, clinicians and industry) must develop, advocate, and continuously revise strict best-practice guidelines for acquisition and analysis of IBs.
  *Rationale.* Editors, referees and readers should be able to evaluate whether a study of an IB is compliant with best-practice, or whether investigators have adequately justified any noncompliance with guidelines (for example, rational use of novel image analysis).
3. Single-centre studies should measure IB repeatability, unless compelling reasons exist not to. All therapeutic-intervention studies should report double-baseline repeatability or reproducibility measurements.
  *Rationale.* IB repeatability and reproducibility data used for power calculations must be taken from the centres that will perform the subsequent pharmacodynamic, screening, diagnostic or outcome studies. High-quality repeatability data has beneficial ethical, economic and logistical effects, by reducing sample size, trial duration and trial cost.
4. Multicentre reproducibility across multiple different equipment vendors must be demonstrated in relevant patient populations before phase II or phase III studies are initiated.
  *Rationale.* Results of small-sized outcome studies might not be generalizable to large-sized patient populations examined on a greater variety of devices by staff in non-expert centres.
5. Multicentre studies require a documented lead analysis site to initiate and monitor IB-data quality. Analysis should be performed at one centre (if involving a few sites), or by comparing individual centres to a central read (if involving a large number of sites and if the IB is near to crossing translational gap 2).
  *Rationale.* Variation in IB acquisition and analysis must be minimalized for multicentre studies to optimize likelihood of a validated IB being qualified as fit-for-purpose for clinical use.

### Biological and clinical validation

1. Definitive outcome studies happen late in the IB validation journey. Initial biological validation should be based on graded accumulation of diverse evidence^95^, including early robust imaging–pathology correlations through comprehensive series of studies with patients and relevant animal models (including genetically engineered mouse models, syngeneic models and orthotopic and/or *in-situ* tumours).
  *Rationale.* Biospecimen-derived biomarkers can often be evaluated rapidly against survival rates using biobanked samples. This possibility is uncommon for IBs, unless the IB is derived from images acquired routinely in healthcare.
2. Improved capabilities should be developed for accurate imaging–pathology correlation, including whole-tumour 3D analysis and alternative ‘gold standards’, such as biospecimen-derived readouts.
  *Rationale.* Many IBs have not influenced clinical decision-making because of unclear relationships with tumour biology. Traditional pathology analysis of one or few tissue sections might result in under-sampling of tumours.
3. Multicentre, collaborative and federated efforts are urgently needed to share, store and curate data.
  *Rationale.* Most IB validation necessarily involves new data acquisition. Few centres can recruit enough patients in sufficient time to power large-sized prospective imaging-based studies. Initiatives such as RIDER^102^, led by the US National Cancer Institute, illustrate possible solutions to these problems. Furthermore, image repositories enable rapid evaluation of IBs derived from existing data.
4. All true-negative, false-negative and false-positive data obtained in studies with either animals or humans should be published.
  *Rationale.* Reluctance to publish negative findings results in considerable publication bias and risks overstating the degree of IB validation.

### Qualification

1. Definitive studies linking IB to clinical outcomes must be prospective, in relevant patient populations, and adequately powered. Novel study designs are encouraged.
  *Rationale.* Many IB studies are underpowered for sensitivity, specificity and survival data. Study populations should not be dominated by highly selected patients who are more able to undergo thorough complex imaging protocols than the general patient population.

### Cost-effectiveness

1. New models for funding and regulation should be developed for investigational devices and tracers and/or contrast agents that lack commercial viability as diagnostic products in healthcare systems, but have value in the research setting as IBs.
  *Rationale.* Research related to many valuable IBs involves specialized devices, tracers and contrast agents that are not commercially viable as diagnostic products. Regulatory hurdles are rightly much higher for marketing approval than for investigational use; however, precedent set by the PET community has maintained availability of many investigational tracers for research use even with limited or no marketing prospects.
2. Outcome studies should include health economic considerations. Comparisons of cost per quality-adjusted life year (QALY) should be performed between imaging and competitor biospecimen-based biomarkers.
  *Rationale.* IBs are perceived to be costly; thus, a clear QALY advantage should be demonstrated.

## Footnotes

**SUV_max_** Maximum standardized uptake value is a parameter used routinely in clinical medicine to identify and quantify avidity of tracer uptake in PET studies.

***K*^trans^** Volume transfer co-efficient describes the transendothelial transport of low molecular weight contrast agent from blood vessels into the extravascular-extracellular space by diffusion. This imaging biomarker is commonly used in studies of antivascular agents, and measures a composite of blood flow, vessel permeability and vessel surface area.

**ADC** Apparent diffusion co-efficient is a commonly used imaging biomarker in studies of various therapies, including cytotoxic chemotherapy, targeted agents and radiotherapy. The biomarker is sensitive to motion of water molecules.

**Double baseline studies** Biomarker precision can be assessed by measuring on multiple occasions and calculating the repeatability or reproducibility of the parameter. Typically, this precision is achieved by measuring the biomarker twice at baseline in the absence of any treatment effects.

**Δ*IAUC_60_*** Integrated area under the curve at 60 seconds is a nonspecific imaging biomarker of the tumour vasculature used in studies of antivascular agents. The biomarker measures a composite of blood flow, blood volume, leakage space, vessel permeability and vessel surface area.
