# Supplementary material for: Imaging biomarker roadmap for cancer studies
Source: Nat Rev Clin Oncol. Author manuscript; Available in PMC 2017 Apr 3. (PMC5378302; doi:10.1038/nrclinonc.2016.162)
Supplement: Supplementary information S1 [file NIHMS71926-supplement-Supplementary_information_S1.pdf]

In format provided by O'Connor *et al.* (doi:10.1038/nrclinonc.2016.162)

Supplementary information S1 (table) | Definitions of key terminology

Term	Definition	Contrast with	Example
**3Rs**	The principles of the 3Rs (Replacement, Reduction and Refinement) were developed over 50 years ago[1] as a framework for humane animal research. They form the basis of guidelines on the use of animals in cancer research[2].		
**Assay validation**	See technical validation		
**Binary biomarker test**	A test that reports a positive or negative result for measurement of a biomarker.<br><br>A binary biomarker test is sometimes based on a measurement of an underlying quantitative variable to which a cut-off point is applied, with the test result designated as positive on one side of the cut-off point and negative on the other. For a binary biomarker test based on a categorical variable, positivity is defined by whether or not the measurement belongs to a specified subset of categories.		• In systemic cancer therapy, scintigraphy or echocardiography LVEF is a binary biomarker test for cancer therapeutics related cardiac dysfunction [3]<br>• In loco-regional cancer therapy a biomarker may have a spatially-resolved cut-off point, i.e. spatial locations, if biomarker positive receive different treatment than spatial locations that are biomarker negative. Technetium (^{99m}Tc) tilmanocept is approved for intraoperative detection of sentinel lymph nodes draining a primary tumour in adult patients with breast cancer, melanoma, or localized squamous cell carcinoma of the oral cavity [4]. Biomarker cut-off point is background radioactivity counts +3SD from the mean background count level with background counts determined from tissue at least 200mm distal to the injection site.

Term	Definition	Contrast with	Example
**Biological validation**	The process of establishing the association between the imaging **biomarker** and the underlying physiological, anatomical, or pathological process[5]. Biological validation does not address the ability of the biomarker to forecast clinical outcome.	• Technical (assay) validation<br>• Clinical validation<br>• Surrogacy validation<br>• Qualification	• Drug-induced change in ^{18}F FDG-PET SUV_{max} or median K^{trans} faithfully reflects the change in the underlying biology
**Biomarker**	A defined characteristic that is **measured** as an indicator of normal biological processes, pathogenic processes, or responses to an exposure or intervention, including therapeutic interventions. Molecular, histologic, radiographic, or physiologic characteristics are types of biomarkers. A biomarker is not an assessment of how an individual feels, functions, or survives[6, 7].<br><br>This approach seeks to clarify inconsistency in terminology, since some previous definitions have restricted the scope of biomarkers to biological molecules (see the NCI glossary at http://www.cancer.gov/publications/dictionaries/cancer-terms?cdrid=45618). This narrower definition regards values obtained from imaging and other techniques as measurements of an underlying biomarker, rather than being biomarkers themselves. However, the current FDA/NIH definition[7] takes a broader view.<br><br>In investigational settings where a biomarker has putative use[8], it may be: more or less rigorously defined and measured; more or less accurate as an indicator; and more or less improve the forecast of clinical outcome	• Clinical end point<br>• Sign	• Objective response<br>• Progression-free survival<br>• Change in LVEF<br>• Median K^{trans}
**Biomarker value**	The result of a **biomarker measurement** expressed as a **quantity value**, or **biomarker** classification expressed as a nominal or **ordinal** category		• Objective response may take one of four ordinal categories: CR, PR, SD, PD<br>• LVEF may take any value between 0 and 1<br>• K^{trans} may take any non-negative value

In format provided by O'Connor *et al.* (doi:10.1038/nrclinonc.2016.162)

Term	Definition	Contrast with	Example
**Clinical outcome**	Describes or reflects how a patient feels, functions or survives[7]	• Biomarker	• Some adverse events (common terminology criteria for adverse events) e.g. pain score in activities of daily living[9]<br>• Overall Survival
**Clinical utility**	The conclusion that a given use of a medical product or **biomarker** will lead to a net improvement in health outcome or provide useful information about diagnosis, treatment, management, or prevention of a disease[5, 7, 10].	• Technical (assay) validation<br>• Biological validation<br>• Clinical validation<br>• Qualification	• Changes in FDG-PET SUV_{max} following one cycle of chemotherapy in gastric cancer patients may have clinical utility if patients with changes of > 35% are likely to ultimately benefit from switching to a more intensive chemotherapy regimen (i.e. experience improved survival on the more intensive regimen)[11].
**Clinical validation**	Establishing that the **biomarker** identifies, measures, or predicts the concept of interest[7]. There is not necessarily and evidence that the biomarker improves the forecast of clinical outcome[5, 12].	• Biological validation<br>• Clinical utility<br>• Qualification	• Baseline values or early changes in ^{18}F FDG-PET SUV_{max} or K^{trans} relates to PFS in a small study[13]

Term	Definition	Contrast with	Example
**Companion diagnostic (in a regulatory context)**	A subset of **predictive** biomarkers and **monitoring** biomarkers which are regarded by regulatory authorities[14] as being essential for:<br>• identifying patients who are most likely to benefit from a particular therapeutic product; or<br>• identifying patients likely to be at increased risk for serious side effects as a result of treatment with a particular therapeutic product; or<br>• monitoring response to treatment with a particular therapeutic product for the purpose of adjusting treatment to achieve improved safety or effectiveness.<br>FDA defines a companion diagnostic as an '*in vitro* diagnostic device or an imaging tool that provides information that is essential for the safe and effective use of a corresponding therapeutic product'[14]. The companion diagnostic device and its corresponding therapeutic product must be approved or cleared for the use indicated in the therapeutic product labelling	• Predictive biomarker	• Non-oncology example: the FerriScan R_2-MRI Analysis System is approved by FDA[15] as Companion Imaging Diagnostic. This Companion Diagnostic selects which thalassemia patients receive deferasirox<br>• FR+ (folate receptor positive status assessed ^{99m}Tc-etarfolatide) was recommended by the EMA[16] for approval as a companion imaging diagnostic for platinum-resistant ovarian cancer patients treated with vintafolide[17], based on phase II data[18]. Recommendation was conditional on the outcome of the phase III PROCEED trial, which was negative [19]
**Cut-off point**	A specific value used to create a **binary biomarker test** based on a quantitative measurement. At one side of the cut-off point patients are "biomarker positive" and at the other side, biomarker "negative". The cut-off point controls the **sensitivity** and **specificity** of the **binary biomarker test**. Occasionally more than one cut-off point may be used e.g. two cut-off points would produce a ternary biomarker test.		• LVEF=53% is a cut-off point for CTRCD[3]
**Devalidation**	Interpretation of evidence leading to the conclusion that a **biomarker** is unsuitable for a specified purpose[20, 21].	• Validation	
**Diagnostic biomarker**	A **biomarker** used to identify individuals with the disease or condition of interest or to define a subset of the disease[7]	• Predictive biomarker<br>• Prognostic biomarker	• Radiolabelled somatostatin receptor analogues (including ^{111}In-pentetreotide octreotide SPECT)[22] and ^{68}Ga-dotatate (PET-CT)[23] identify neuroendocrine tumour sites

Term	Definition	Contrast with	Example
**Image**	An array of values varying in two or more spatial dimensions derived from analysis of an **imaging signal** and corresponding to an array of spatial locations in the body	• Imaging device<br>• Imaging modality<br>• Imaging technique<br>• Imaging signal	• Clinically available modalities include plain films, mammogram, CT, SPECT and PET, MRI and Ultrasound<br>• Other modalities include optical, near-infrared and photo-acoustic methods
**Imaging Biomarker**	A spatially delineated **biomarker** derived from **measurements** made on an image	• Imaging device<br>• Imaging modality<br>• Imaging technique<br>• Imaging signal	• Clinical TNM stage<br>• Objective response<br>• LVEF<br>• Median K^{trans}<br>• ^{18}F FDG-PET SUV_{max}
**Imaging Device**	A specific make and model of medical device (hardware and/or software) which is a) regulated in the USA by FDA Center for Devices and Radiological Health by 510(k) clearance[24] and in the EU by CE marking, and b) employed in the creation of an **image** or an **imaging biomarker**	• Imaging modality<br>• Imaging technique<br>• Imaging signal	• Clinically approved CT, SPECT and PET, MRI or ultrasound scanner<br>• Tumour segmentation software approval by the FDA[25]
**Imaging Modality**	A category of **imaging device**, characterised by a distinct physical principle	• Imaging technique<br>• Imaging device<br>• Imaging signal	• CT<br>• DXA<br>• Mammography<br>• MRI<br>• PET<br>• SPECT<br>• Ultrasonography
**Imaging Signal**	Energy which is transmitted, emitted, or modulated by a body and detected by an **imaging device**	• Imaging biomarker<br>• Imaging device<br>• Imaging technique	• List mode<br>• Free induction decay
**Imaging Technique**	A specific way of deploying an **imaging modality** to produce an **image**	• Imaging modality<br>• Imaging signal<br>• Imaging device	• DWI<br>• Dynamic ^{18}F FDG-PET

Term	Definition	Contrast with	Example
**Measurement**	The process of experimentally obtaining one or more **quantity values** that can be reasonably attributed to a **quantity**[26]. Measurements are obtained using a **test, tool or instrument**[7].		• Performing a CT scan on a patient, displaying the image, and using image segmentation software to measure tumor volume in ml
**Monitoring biomarker**	A biomarker measured serially and used to detect a change in the degree or extent of disease. Monitoring biomarkers may also be used to indicate toxicity or assess safety, or to provide evidence of exposure, including exposures to medical products [7].	• Response biomarker	• Left ventricular ejection fraction [3] is an imaging biomarker used to monitor cardiotoxicity in patients treated with trastuzumab [27]
**Morphologic biomarker**	A **biomarker** that **measures** the size or shape of a macroscopic structure in the body	• Pharmacodynamic biomarker	• 1D size<br>• Tumor volume
**Ordinal biomarker**	A **biomarker** which is expressed as a category on a scale containing three or more labelled categories that can be meaningfully ordered[28]. An ordinal biomarker with just two categories is a **binary biomarker**	• Quantitative biomarker	• ACR BIRADS breast composition is an ordinal biomarker whose scale contains five ordered labelled categories [29]
**Pharmacodynamic Biomarker**	Most commonly used to refer to a **biomarker** measuring the duration and magnitude of the pharmacologic response observed relative to the concentration of a drug at its active site in the patient. More generally, this can describe "what the drug does to the body" before tumour size is affected [30]	• Pharmacokinetic biomarker<br>• Morphological biomarker	• K^{trans} is a DCE-MRI pharmacodynamic biomarker which shows a dose-dependent reduction in tumors following treatment with certain tyrosine kinase inhibitors such as cediranib [31] and vatalanib [32]<br>• AUC is a DCE-US biomarker which shows a dose-dependent reduction in tumors following treatment with certain tyrosine kinase inhibitors such as sunitinib [33]
**Pharmacologic audit trail**	The concept that the overarching drug development hypothesis (that the investigational treatment has sufficient clinical benefit and lack-of-harm in a particular population) can be broken down into a sequence of sub-hypotheses (that the drug: reaches its target; engages its target; modulates the target pathway; alters cell phenotype; modulates local physiology; and elicits disease-relevant structural change) [30].	• Proof-of-concept<br>• Proof-of-mechanism<br>• Proof-of-principle	

Term	Definition	Contrast with	Example
**Pharmacokinetic biomarker**	A **biomarker** that measures directly the pharmacokinetics (absorption, distribution, metabolism, excretion) of a drug, i.e. "what the body does to the drug"[30]. Most commonly, the **imaging signal** arises directly from the drug molecule itself	• Pharmacodynamic Biomarker	• FDA requires that PK be verified by imaging before a therapeutic dose of the radio-therapeutic drug ^{131}I-tositumomab can be given
**Predictive biomarker**	A **biomarker** used to identify individuals who are more likely to experience a favourable or unfavourable effect from a specific intervention or exposure[7]	• Companion diagnostic<br>• Prognostic biomarker	• ^{111}In pentetreotide uptake predicts efficacy of octreotide<br>• In phase II, FR+ (folate receptor positive status assessed ^{99m}Tc-etarfolatide) predicts how well platinum-resistant ovarian cancer patients will respond to vintafolide[18]
**Prentice criterion**	A requirement that treatment effects on the surrogate endpoint must fully capture treatment effects on the definitive endpoint (e.g. OS) [34]		• None
**Prognostic biomarker**	A **biomarker** used to identify likelihood of a clinical event, disease recurrence or progression[6, 7].<br><br>A **biomarker** that provides information about the natural history of the cancer in an individual in the absence of treatment [35]. However, if the biomarker is also predictive in the context of a particular treatment, then effects of that treatment may be confused with prognostic ability of the marker when examined in patients receiving that treatment. For this reason, some prefer to define prognostic biomarkers as those which predict outcome in the absence of treatment or under some standard therapy that all patients are likely to receive.	• Predictive Biomarker	• Bone Scan Index, an imaging biomarker of the fraction of the total skeletal mass affected by metastatic disease, is a prognostic biomarker in prostate cancer [36] with FDA approved software [37]
**Proof-of-concept**	In drug development[30], following positive **proof-of-principle**, a positive test of the hypothesis that the investigational drug affects the disease in human subjects	• Proof-of-mechanism<br>• Proof-of-principle	• Objective response

Term	Definition	Contrast with	Example
**Proof-of-mechanism**	In drug development[30], a positive test of the hypothesis that the investigational drug engages its putative molecular target in human subjects	• Proof-of-concept<br>• Proof-of-principle	• The total-choline signal in MRS may provide a proof-of-mechanism biomarker in early-phase trials of choline kinase inhibitors[38]<br>• Change in ^{18}F-FDG PET SUV_{max} demonstrates target inhibition and may provide a proof-of-mechanism biomarker in early-phase trials of AKT inhibitors[39]
**Proof-of-principle**	In drug development[30], following positive **proof-of-mechanism**, a positive test of the hypothesis that the investigational drug affects the putative disease mechanism in human subjects with that disease	• Proof-of-concept<br>• Proof-of-mechanism	• Change in K^{trans} demonstrated modulation of tumor perfusion/permeability and provided a proof-of-mechanism biomarker in early-phase trials of cediranib[31] and vatalanib[32]
**Qualification**	1. Evidentiary process of linking a **biomarker** with biological processes and **clinical endpoints** for a specific use[40, 41]. This may be in a variety of contexts, including a randomized prospective clinical trial or through the prospective analysis of large retrospective patient cohorts.<br>2. The regulatory sense describes a different process – it is a conclusion, based on a formal regulatory process[42], that within the stated context of use, a medical product development tool can be relied upon to have a specific interpretation and application in medical product development and regulatory review[7].	• Technical (assay) validation<br>• Biological validation<br>• Clinical validation<br>• Surrogacy validation<br>• Qualification	• Demonstrating a sufficiently rigorous volumetric CT-based biomarker to allow individual patients in clinical settings to switch treatments sooner if they are no longer responding to their current regimens, and reduce the costs of evaluating investigational new drugs to treat lung cancer[43]
**Qualitative imaging biomarker**	A **biomarker** that cannot be expressed as a **quantity value**[26]. All ordinal biomarkers are examples.	• Qualitative Imaging Biomarker	• Pathological grading systems<br>• Clinical TNM Stage
**Quantitative imaging biomarker**	A **biomarker** whose magnitude is expressed as a **quantity value**. A quantitative imaging biomarker is an objective characteristic derived from an *in vivo* image as indicators of normal biological processes, pathogenic processes or a response to a therapeutic intervention[26]	• Ordinal Biomarker<br>• Qualitative Imaging Biomarker	• Tumour volume, derived from volumetric CT

Term	Definition	Contrast with	Example
**Quantity value**	A number and units together that express the magnitude of a quantity[26]		• Tumour volume expressed in ml
**Repeatability**	A measure of the extent to which a test conducted multiple times on the same subject, in the same lab, using the same equipment, by the same operator, over a short period of time, should give the same result[28]	• Reproducibility	• The biomarker test-retest coefficient of variation In a study where the imaging biomarker is measured twice in quick succession by the same investigator using the same imaging device in the same hospital
**Reproducibility**	A measure of the extent to which a test conducted multiple times in different labs, using different equipment, by different operators, or over different periods of time, should give comparable results[28]	• Repeatability	• The biomarker test-retest coefficient of variation In a study where the imaging biomarker is measured by different investigators using different imaging devices in different hospitals
**Response biomarker**	A **biomarker** whose change after therapy forecasts benefit or lack of benefit from the therapy. In the research setting, response biomarkers are used to assess the effects of investigational therapies (**pharmacologic audit trail**). In healthcare, response biomarkers are used in the trial-of-therapy setting to identify individuals who are likely to experience a favourable or unfavourable effect from the therapy, so that the biomarker <u>change</u> may be regarded as an instance of **predictive biomarker**.	• Monitoring biomarker<br>• Pharmacological biomarker	• Objective response following chemotherapy in advanced colorectal cancer predicts survival[44]<br>• AUC decrease in DCE-US following treatment with anti-angiogenic drugs predicts PFS and OS[33]
**Safety biomarker**	A **biomarker** used to indicate the presence or extent of toxicity related to an intervention or exposure[7]		• Left ventricular ejection fraction[3] is an imaging biomarker used to monitor cardiotoxicity in patients treated with trastuzumab[27]<br>• Bone mineral density is an imaging biomarker of enhanced fracture risk in patients with breast cancer treated with aromatase inhibitors[45]

Term	Definition	Contrast with	Example
**Sensitivity (of an imaging biomarker)**	Measure of how often a **binary biomarker test** correctly indicates presence of a particular characteristic (e.g. disease) in patients that truly have the characteristic.<br><br>Biomarker sensitivity is the number of true positive results divided by the number of true positive plus false negative results	• Biomarker specificity	• Screening categorical biomarker based on morphology and contrast-enhanced MRI has 77% sensitivity and 81% specificity for detecting breast cancers[46]
**Sign**	An abnormality indicative of disease, discoverable on examination of the patient. A sign that is defined and measured becomes a **biomarker**.	• Biomarker	• Ground glass opacity
**Specificity (of an imaging biomarker)**	Measure of how often a **binary biomarker test** correctly indicates absence of a particular characteristic (e.g. disease) in patients that truly do not have the characteristic.<br>Biomarker specificity is the number of true negative results divided by the number of true negative plus false positive results	• Biomarker sensitivity	• Screening categorical biomarker based on morphology and contrast-enhanced MRI has 77% sensitivity and 81% specificity for detecting breast cancers[46]
**Surrogate endpoint**	An end point that is used in clinical trials as a substitute for a direct measure of how a patient feels, functions, or survives. A surrogate end point does not measure the clinical benefit of primary interest in and of itself, but rather is expected to predict that clinical benefit or harm based on epidemiologic, therapeutic, pathophysiologic, or other scientific evidence[6].		• Objective response rate has been used by the FDA[47] as a reasonably likely surrogate end point in the accelerated approval of brentuximab, crizotinib, vismodegib, carfilzomib, pomalidomide, ibrutinib, siltuximab, ceritinib, belinostat, idelalisib, olaparib and nivolumab between January 2010 to December 2014
**Technical (assay) validation**	The process of establishing the **accuracy**, **repeatability**, **reproducibility** and lack of **bias** of a biomarker measurement under conditions which are ethical and legal in relevant jurisdictions	• Biological validation<br>• Clinical validation<br>• Surrogacy validation<br>• Qualification	• Technical validation of ^{18}F FDG-PET signal is mandated by EANM for accreditation
**Test, tool or instrument**	An assessment system comprising three essential components: 1) materials for measurement; 2) an assay for obtaining the measurement; and 3) method and/or criteria for interpreting those measurements[7]		

Term	Definition	Contrast with	Example
**Translational gaps**<br>**Translational blocks**	In the UK, the Cooksey report[48] identified two key gaps in the translation of health research:<br>1. translating ideas from basic and clinical research into the development of new products and approaches to treatment of disease and illness; and<br>2. implementing those new products and approaches into clinical practice.<br>In the USA, the Institute of Medicine's Clinical Research Roundtable[49] identified two "translational blocks" in the clinical research enterprise:<br>1. the transfer of new understandings of disease mechanisms gained in the laboratory into the development of new methods for diagnosis, therapy, and prevention and their first testing in humans<br>2. the translation of results from clinical studies into everyday clinical practice and health decision making		Gap 1 crossed:<br>• The imaging biomarker is a primary or secondary end point in a therapeutic trial<br>Gap 2 crossed:<br>• New drug application to FDA for a PET or SPECT tracer

References

1. Russell, W.M.S. & Burch, R.L. The Principles of Humane Experimental Technique (Methuen, London, 1959).
2. Workman, P., Aboagye, E.O., Balkwill, F., Balmain, A., Bruder, G., Chaplin, D.J. et al. Guidelines for the welfare and use of animals in cancer research. *Br J Cancer* **102**, 1555-77 (2010).
3. Plana, J.C., Galderisi, M., Barac, A., Ewer, M.S., Ky, B., Scherrer-Crosbie, M. et al. Expert consensus for multimodality imaging evaluation of adult patients during and after cancer therapy: a report from the American Society of Echocardiography and the European Association of Cardiovascular Imaging. *Eur Heart J Cardiovasc Imaging* **15**, 1063-93 (2014).
4. FDA. http://www.fda.gov/NewsEvents/Newsroom/PressAnnouncements/ucm343525.htm (accessed 30th November 2015).
5. McShane, L.M. & Hayes, D.F. Publication of tumor marker research results: the necessity for complete and transparent reporting. *J Clin Oncol* **30**, 4223-32 (2012).
6. Biomarkers Definitions Working Group. Biomarkers and surrogate endpoints: preferred definitions and conceptual framework. *Clin Pharmacol Ther* **69**, 89-95 (2001).
7. FDA-NIH Biomarker Working Group. BEST (Biomarkers, EndpointS, and other Tools) Resource. http://www.ncbi.nlm.nih.gov/books/NBK326791 (accessed 4th February 2016).

8. Lesko, L.J. & Atkinson, A.J., Jr. Use of biomarkers and surrogate endpoints in drug development and regulatory decision making: criteria, validation, strategies. *Annu Rev Pharmacol Toxicol* **41**, 347-66 (2001).
9. O'Toole, J.A., Ferguson, C.M., Swaroop, M.N., Horick, N., Skolny, M.N., Brunelle, C.L. et al. The impact of breast cancer-related lymphedema on the ability to perform upper extremity activities of daily living. *Breast Cancer Res Treat* **150**, 381-8 (2015).
10. Hayes, D.F., Allen, J., Compton, C., Gustavsen, G., Leonard, D.G., McCormack, R. et al. Breaking a vicious cycle. *Sci Transl Med* **5**, 196cm6 (2013).
11. https://clinicaltrials.gov/ct2/show/NCT02485834 (accessed 19th February 2016).
12. Parkinson, D.R., McCormack, R.T., Keating, S.M., Gutman, S.I., Hamilton, S.R., Mansfield, E.A. et al. Evidence of clinical utility: an unmet need in molecular diagnostics for patients with cancer. *Clin Cancer Res* **20**, 1428-44 (2014).
13. De Bruyne, S., Van Damme, N., Smeets, P., Ferdinande, L., Ceelen, W., Mertens, J. et al. Value of DCE-MRI and FDG-PET/CT in the prediction of response to preoperative chemotherapy with bevacizumab for colorectal liver metastases. *Br J Cancer* **106**, 1926-33 (2012).
14. FDA. http://www.fda.gov/MedicalDevices/ProductsandMedicalProcedures/InVitroDiagnostics/ucm407297.htm (accessed 8th October 2015).
15. FDA. http://www.fda.gov/MedicalDevices/ProductsandMedicalProcedures/InVitroDiagnostics/ucm301431.htm (accessed 8th October 2015).
16. European Medicines Agency Committee for Medicinal Products for Human Use. Summary of opinion: Folcepri etarfolatide EMA/CHMP/139146/2014 http://www.ema.europa.eu (Accessed 30th November 2015).
17. European Medicines Agency Committee for Medicinal Products for Human Use. Summary of opinion (initial authorisation): Vynfinit vintafolide EMA/CHMP/138539/2014 http://www.ema.europa.eu (Accessed 30th November 2015).
18. Morris, R.T., Joyrich, R.N., Naumann, R.W., Shah, N.P., Maurer, A.H., Strauss, H.W. et al. Phase II study of treatment of advanced ovarian cancer with folate-receptor-targeted therapeutic (vintafolide) and companion SPECT-based imaging agent (99mTc-etarfolatide). *Ann Oncol* **25**, 852-8 (2014).
19. Endocyte Press Release. http://investor.endocyte.com/releasedetail.cfm?ReleaseID=844838 (accessed 8th October 2015).
20. Barker, A., Kettle, J.G., Nowak, T. & Pease, J.E. Expanding medicinal chemistry space. *Drug Discov Today* **18**, 298-304 (2013).
21. Blagg, J. & Workman, P. Chemical biology approaches to target validation in cancer. *Curr Opin Pharmacol* **17**, 87-100 (2014).
22. Kam, B.L., Teunissen, J.J., Krenning, E.P., de Herder, W.W., Khan, S., van Vliet, E.I. et al. Lutetium-labelled peptides for therapy of neuroendocrine tumours. *Eur J Nucl Med Mol Imaging* **39 Suppl 1**, S103-12 (2012).
23. Ambrosini, V., Nanni, C. & Fanti, S. The use of gallium-68 labeled somatostatin receptors in PET/CT imaging. *PET Clin* **9**, 323-9 (2014).
24. FDA. http://www.fda.gov/MedicalDevices/DeviceRegulationandGuidance/Overview/default.htm (accessed 8th October 2015).
25. Columbia University http://techventures.columbia.edu/news-and-events/latest-news/tumor-segmentation-software-receives-510k-clearance-fda (accessed 8th October 2015).
26. Kessler, L.G., Barnhart, H.X., Buckler, A.J., Choudhury, K.R., Kondratovich, M.V., Toledano, A. et al. The emerging science of quantitative imaging biomarkers terminology and definitions for scientific studies and regulatory submissions. *Stat Methods Med Res* **24**, 9-26 (2015).
27. HERCEPTIN® (trastuzumab). Highlights of Prescribing Information (2010). www.accessdata.fda.gov/drugsatfda_docs/label/2010/103792s5256lbl.pdf (accessed 8th October 2015).
28. ISO. 3534-2:2006 Statistics – Vocabulary and symbols – Part 2: Applied statistics (accessed 30th November 2015).
29. American College of Radiology (ACR). Breast imaging reporting and data system atlas (BI-RADS atlas) (American College of Radiology, Reston, VA, 2013).
30. Workman, P., Aboagye, E.O., Chung, Y.L., Griffiths, J.R., Hart, R., Leach, M.O. et al. Minimally invasive pharmacokinetic and pharmacodynamic technologies in hypothesis-testing clinical trials of innovative therapies. *J Natl Cancer Inst* **98**, 580-98 (2006).

31. Drevs, J., Siegert, P., Medinger, M., Mross, K., Strecker, R., Zirrgiebel, U. et al. Phase I clinical study of AZD2171, an oral vascular endothelial growth factor signaling inhibitor, in patients with advanced solid tumors. *J Clin Oncol* **25**, 3045-54 (2007).
32. Morgan, B., Thomas, A.L., Drevs, J., Hennig, J., Buchert, M., Jivan, A. et al. Dynamic contrast-enhanced magnetic resonance imaging as a biomarker for the pharmacological response of PTK787/ZK 222584, an inhibitor of the vascular endothelial growth factor receptor tyrosine kinases, in patients with advanced colorectal cancer and liver metastases: results from two phase I studies. *J Clin Oncol* **21**, 3955-64 (2003).
33. Lassau, N., Bonastre, J., Kind, M., Vilgrain, V., Lacroix, J., Cuinet, M. et al. Validation of dynamic contrast-enhanced ultrasound in predicting outcomes of antiangiogenic therapy for solid tumors: the French multicenter support for innovative and expensive techniques study. *Invest Radiol* **49**, 794-800 (2014).
34. Prentice, R.L. Surrogate endpoints in clinical trials: definition and operational criteria. *Stat Med* **8**, 431-40 (1989).
35. Oldenhuis, C.N., Oosting, S.F., Gietema, J.A. & de Vries, E.G. Prognostic versus predictive value of biomarkers in oncology. *Eur J Cancer* **44**, 946-53 (2008).
36. Sabbatini, P., Larson, S.M., Kremer, A., Zhang, Z.F., Sun, M., Yeung, H. et al. Prognostic significance of extent of disease in bone in patients with androgen-independent prostate cancer. *J Clin Oncol* **17**, 948-57 (1999).
37. FDA. http://www.accessdata.fda.gov/cdrh_docs/pdf12/K122205.pdf (accessed 30th November 2015).
38. Kumar, M., Arlauckas, S.P., Saksena, S., Verma, G., Ittyerah, R., Pickup, S. et al. Magnetic resonance spectroscopy for detection of choline kinase inhibition in the treatment of brain tumors. *Mol Cancer Ther* **14**, 899-908 (2015).
39. Sarker, D., Ang, J.E., Baird, R., Kristeleit, R., Shah, K., Moreno, V. et al. First-in-human phase I study of pictilisib (GDC-0941), a potent pan-class I phosphatidylinositol-3-kinase (PI3K) inhibitor, in patients with advanced solid tumors. *Clin Cancer Res* **21**, 77-86 (2015).
40. Cancer Research UK. http://www.cancerresearchuk.org/sites/default/files/biomarker_project_award_guidelines_june2014.pdf (accessed 5 February 2016).
41. Micheel, C.M. & Ball, J.R. Evaluation of Biomarkers and Surrogate Endpoints in Chronic Disease (National Academy of Sciences, Washington, 2010).
42. FDA. http://www.fda.gov/drugs/developmentapprovalprocess/drugdevelopmenttoolsqualificationprogram/ucm284076.htm (accessed 30th November 2015).
43. Buckler, A.J., Mozley, P.D., Schwartz, L., Petrick, N., McNitt-Gray, M., Fenimore, C. et al. Volumetric CT in lung cancer: an example for the qualification of imaging as a biomarker. *Acad Radiol* **17**, 107-15 (2010).
44. Buyse, M., Thirion, P., Carlson, R.W., Burzykowski, T., Molenberghs, G. & Piedbois, P. Relation between tumour response to first-line chemotherapy and survival in advanced colorectal cancer: a meta-analysis. Meta-Analysis Group in Cancer. *Lancet* **356**, 373-8 (2000).
45. ARIMIDEX® (anastrozole). Highlights of prescribing information. (2010) http://www.accessdata.fda.gov/drugsatfda_docs/label/2011/020541s026lbl.pdf.
46. Leach, M.O., Boggis, C.R., Dixon, A.K., Easton, D.F., Eeles, R.A., Evans, D.G. et al. Screening with magnetic resonance imaging and mammography of a UK population at high familial risk of breast cancer: a prospective multicentre cohort study (MARIBS). *Lancet* **365**, 1769-78 (2005).
47. FDA. http://www.fda.gov/downloads/NewsEvents/Testimony/UCM445375.pdf (accessed 30th November 2015).
48. Cooksey, D. A review of UK health research funding (TSO, Norwich, 2006).
49. Sung, N.S., Crowley, W.F., Jr., Genel, M., Salber, P., Sandy, L., Sherwood, L.M. et al. Central challenges facing the national clinical research enterprise. *Jama* **289**, 1278-87 (2003).
