# Supplementary material for: Imaging biomarker roadmap for cancer studies
Source: Nat Rev Clin Oncol. Author manuscript; Available in PMC 2017 Apr 3. (PMC5378302; doi:10.1038/nrclinonc.2016.162)
Supplement: Supplementary information S2 [file NIHMS71926-supplement-Supplementary_information_S2.pdf]

In format provided by O'Connor *et al.* (doi:10.1038/nrclinonc.2016.162)

Supplementary information S2 (table) | Important factors to consider when validating and qualifying imaging biomarkers (IBs)

The fundamental differences between IBs and biospecimen biomarkers limit the relevance of adapting biospecimen biomarker roadmaps to the development of IBs (1).

Imaging devices, contrast agents/tracers and software

Researchers performing IB research use many different imaging devices (scanners), purchased from different vendors. Scanners are designed and sold to healthcare providers to produce images which diagnostic radiologists, nuclear medicine physicians and other clinicians interpret, often without quantifying the image, other than defining lesion presence and measuring lesion size. Further, imaging devices are nearly always designed, maintained and approved for *qualitative* radiological assessment (2). Innovation is largely driven by competition to improve image quality, improve the user interface and reduce scan time and to create products that distinguish one manufacturer from its competitors. Vendors and purchasers may pay some attention to how innovation may substantially alter IB quantification or standardisation, but these concerns remain secondary to qualitative radiological needs. The result is that measurements and dependent IBs may become vendor-specific rather than vendor-neutral.

These issues can hinder IB acquisition and analysis standardisation (2). For example, in MRI, nominally similar b values involve field gradient values that vary substantially within and between scanners, due to lack of calibration compared with CT, PET and mass spectroscopy (3). Consequently, IBs may give numerically different values on different scanners which hinder comparison between data from different centres (4-6) and also complicates the design and interpretation of multicentre studies. In distinction, biospecimen biomarkers are often analysed using an *in vitro* diagnostic device that was designed and maintained (and in some cases granted regulatory approval) for the specific purpose of measuring the specified analyte (7).

Some IBs have an additional complication compared to biospecimen biomarkers as they often incorporate contrast agents to derive the biomarker, the choice of which can be limited due to the market size required for commercial viability for a different purpose (i.e. as a clinical diagnostic) . This can complicate translation of the IB: for example some PET agents based on short-lived radionuclides such as ^{11}C will depend on the availability of a cyclotron to provide the tracer (8). In MRI, gadolinium-based contrast agents are designed and marketed to improve qualitative diagnosis. Low molecular weight agents such as gadopentetate (MW548) have regulatory approval for this purpose, but some animal studies show advantages for high molecular weight agents such as gadomelitol (MW6474) for deriving IBs of perfusion (9). Although gadomelitol was taken into clinical development, it did not prove sufficiently attractive as a commercial diagnostic product despite its appeal in providing a biomarker (10).

Most IBs require post-acquisition analysis. Many commonly studied IBs (e.g. ADC, the family of K^{trans} and related perfusion biomarkers derived from either CT or MRI and ^{18}F-fluorothymidine PET) continue to be measured and analysed in disparate ways by different laboratories using a wide range of software systems, despite attempts to standardise and harmonise these methods (2, 3, 11-14). Lack of standardisation has impaired multicentre reproducibility for many IB measurements, which has made IB translation and meta-analysis problematic (5). For example, identical data can have order of magnitude differences in numeric values for K^{trans} depending on choice of tracer kinetic model (with differing underlying assumptions) and the software used (15). This is the major reason why absolute values of K^{trans} have not been qualified as prognostic or predictive IBs (Box 6). Even if software packages are commercialised, they are subject to instability following upgrades that can alter biomarker values significantly (16).

Tumor sampling

Many biospecimen biomarkers represent a molecular entity, or "an analyte" (17) that can be measured (18), although some such as tumor grade do not. Some IBs are associated with a specific molecular entity or process ("molecular imaging") which they may purport to directly quantify. However, many IBs lack any associated analyte that could be isolated and assayed (e.g. TNM, ADC, K^{trans} or tumor texture metrics).

Further, biospecimen analytes are measured using an *in vitro* diagnostic assay in a process quite separate from collection of the sample from the patient (19). Biospecimen samples can be split following collection, allowing multiple laboratories to compare reproducibility between instruments, between supposedly similar assays, and between the different laboratories (20). In distinction, the IB arises from a signal detected at the moment when the patient is coupled to the *in vivo* imaging device, quite unlike the relationship of patient to machine seen with *in vitro* biochemical assays (1). Although raw scanner data can be analyzed at different laboratories, acquisition-specific sources of bias and variance are already embedded irreversibly in this work-flow.

A significant advantage of IBs is their ability to provide serial non-invasive measurements (unlike most tissue based assays). In addition, they can distinguish, characterize and track whole and/or multiple lesions within a subject (inter-tumoral heterogeneity) and also quantify intra-tumoral heterogeneity within a lesion (21). These factors are key indications for using imaging in cancer research. Tumor heterogeneity can be a major confounding factor in non-imaging studies where biomarkers can be at extremes of either under-sampling a single lesion (e.g. where biospecimen biomarkers derived from biopsy based on gene mutation or receptor status may be discordant between primary and metastatic lesions in the same individual (22)) or integrating samples from the entire patient at the expense of losing lesion specific information (e.g. circulating free DNA, circulating tumor cells or serum proteome). Investigators have greater control over tumor sampling in IB studies, where sampling can be restricted to part of a tumor, the whole tumor, comparison between multiple tumors within one patient, or total disease burden (21). While an advantage for IBs, this means technical and biological validation studies must be tailored to suit the sampling method employed for each imaging study.

Resources, patient commitment and staff requirements

Some IB studies can proceed on standard of care images (23). Many IB validation studies require acquisition of new data because appropriate raw images do not exist already in an image bank or repository. Recruiting even moderate numbers of patients to imaging studies can be challenging, costly and may require substantial patient commitment without therapeutic benefit.

Some CT, MRI and PET research protocols require intravenous access for contrast or radiopharmaceutical administration. Studies may require multiple examinations and scan time may last up to several hours (5). Some modalities can be uncomfortable, noisy (MRI) and require invasive procedures (e.g. arterial lines to provide input functions and allow metabolite analysis in some early validation studies in PET). Other modalities involve ionizing radiation. However, not all IB studies have as many practical issues; for example DCE-US protocols can be more rapid, cheaper and require less patient commitment (14).

The requirements of IB studies must be compared to those found with biospecimen biomarker studies. For example, if only one biopsy is performed or if biofluid samples are collected along with samples taken for routine clinical care, then this may be seen as attractive relative to imaging-based studies. Biopsies do however generally carry greater clinical risk than most imaging methods. Limited recruitment may lead to highly selected patient groups for initial validation studies that may be unrepresentative of IB performance in large populations (2).

Developing a program of clinical studies to validate and qualify the most promising IBs takes considerable time and resource. This is quite different to developing a biospecimen biomarker from biobanked samples (2) or the specific case developing an IB from readily available clinical scan data such as radiotherapy planning (24) or diagnostic CT scans (25, 26). Qualification of an IB for a particular use (e.g. the role of PET ^{18}F-FDG SUV_{max} in patient selection in an adaptive phase II study) (27) may take many years and will be expensive.

Finally, IBs depend enormously on the performance of clinicians (radiologists, nuclear medicine physicians and others), technicians (including radiographers) and scientists. This is well appreciated for ultrasound (28), but is true for all imaging modalities. While biospecimen biomarkers are also vulnerable to "pre-analytical" factors, the scope for human error and impact of human variability are likely to be high in imaging studies with multiple acquisition and analysis steps. Unfortunately, mistakes and human variability associated with imaging are often not documented and can seldom be rectified retrospectively.

References

1. Waterton JC, Pylkkanen L. Qualification of Imaging Biomarkers for Oncology Drug Development. Eur J Cancer. 2012;48(4):409-15.
2. Dorfman GS, Sullivan DC, Schnall MD, Matrisian LM. The Translational Research Working Group developmental pathway for image-based assessment modalities. Clin Cancer Res. 2008 Sep 15;14(18):5678-84.
3. Padhani AR, Liu G, Koh DM, Chenevert TL, Thoeny HC, Takahara T, et al. Diffusion-weighted magnetic resonance imaging as a cancer biomarker: consensus and recommendations. Neoplasia. 2009 Feb;11(2):102-25.
4. Patterson DM, Padhani AR, Collins DJ. Technology Insight: water diffusion MRI--a potential new biomarker of response to cancer therapy. Nat Clin Pract Oncol. 2008 Apr;5(4):220-33.
5. O'Connor JP, Jackson A, Parker GJ, Roberts C, Jayson GC. Dynamic contrast-enhanced MRI in clinical trials of antivascular therapies. Nat Rev Clin Oncol. 2012;9(3):167-77.
6. Shankar LK. The clinical evaluation of novel imaging methods for cancer management. Nat Rev Clin Oncol. 2012 Dec;9(12):738-44.
7. Micheel C, Nass SJ, Omenn GS, Institute of Medicine Committee on the Review of Omics-Based Tests for Predicting Patient Outcomes in Clinical Trials. Evolution of translational omics: lessons learned and the path forward. Washington: National Academy of Sciences; 2012.
8. Avril N, Propper D. Functional PET imaging in cancer drug development. Future oncology (London, England). 2007 Apr;3(2):215-28.
9. Bradley DP, Tessier JL, Checkley D, Kuribayashi H, Waterton JC, Kendrew J, et al. Effects of AZD2171 and vandetanib (ZD6474, Zactima) on haemodynamic variables in an SW620 human colon tumour model: an investigation using dynamic contrast-enhanced MRI and the rapid clearance blood pool contrast agent, P792 (gadomelitol). NMR Biomed. 2008 Jan;21(1):42-52.
10. Barnes SL, Whisenant JG, Loveless ME, Yankeelov TE. Practical Dynamic Contrast Enhanced MRI in Small Animal Models of Cancer: Data Acquisition, Data Analysis, and Interpretation. Pharmaceutics. 2012 Sep 19;4(3):442-78.
11. Boellaard R, Delgado-Bolton R, Oyen WJ, Giammarile F, Tatsch K, Eschner W, et al. FDG PET/CT: EANM procedure guidelines for tumour imaging: version 2.0. Eur J Nucl Med Mol Imaging. 2015 Feb;42(2):328-54.
12. Leach MO, Brindle KM, Evelhoch JL, Griffiths JR, Horsman MR, Jackson A, et al. The assessment of antiangiogenic and antivascular therapies in early-stage clinical trials using magnetic resonance imaging: issues and recommendations. Br J Cancer. 2005 May 9;92(9):1599-610.
13. Miles KA, Lee TY, Goh V, Klotz E, Cuenod C, Bisdas S, et al. Current status and guidelines for the assessment of tumour vascular support with dynamic contrast-enhanced computed tomography. Eur Radiol. 2012 Jul;22(7):1430-41.
14. Lassau N, Chapotot L, Benatsou B, Vilgrain V, Kind M, Lacroix J, et al. Standardization of dynamic contrast-enhanced ultrasound for the evaluation of antiangiogenic therapies: the French multicenter Support for Innovative and Expensive Techniques Study. Invest Radiol. 2012 Dec;47(12):711-6.

15. Ng CS, Wei W, Bankson JA, Ravoori MK, Han L, Brammer DW, et al. Dependence of DCE-MRI biomarker values on analysis algorithm. PLoS One. 2015;10(7):e0130168.
16. Mazzei MA, Squitieri NC, Sani E, Guerrini S, Imbriaco G, Di Lucia D, et al. Differences in perfusion CT parameter values with commercial software upgrades: a preliminary report about algorithm consistency and stability. Acta Radiol. 2013 Sep;54(7):805-11.
17. McShane LM, Hunsberger S, Adjei AA. Effective incorporation of biomarkers into phase II trials. Clin Cancer Res. 2009 Mar 15;15(6):1898-905.
18. Wagner JA. Strategic approach to fit-for-purpose biomarkers in drug development. Annu Rev Pharmacol Toxicol. 2008;48:631-51.
19. de Bono JS, Ashworth A. Translating cancer research into targeted therapeutics. Nature. 2010 Sep 30;467(7315):543-9.
20. Kraan J, Sleijfer S, Strijbos MH, Ignatiadis M, Peeters D, Pierga JY, et al. External quality assurance of circulating tumor cell enumeration using the CellSearch((R)) system: a feasibility study. Cytometry. 2011 Mar;80(2):112-8.
21. O'Connor JP, Rose CJ, Waterton JC, Carano RA, Parker GJ, Jackson A. Imaging intratumor heterogeneity: role in therapy response, resistance, and clinical outcome. Clin Cancer Res. 2015 Jan 15;21(2):249-57.
22. Bedard PL, Hansen AR, Ratain MJ, Siu LL. Tumour heterogeneity in the clinic. Nature. 2013 Sep 19;501(7467):355-64.
23. Gillies RJ, Kinahan PE, Hricak H. Radiomics: Images Are More than Pictures, They Are Data. Radiology. 2016 Feb;278(2):563-77.
24. Aerts HJ, Velazquez ER, Leijenaar RT, Parmar C, Grossmann P, Cavalho S, et al. Decoding tumour phenotype by noninvasive imaging using a quantitative radiomics approach. Nature communications. 2014;5:4006.
25. Choi H, Charnsangavej C, Faria SC, Macapinlac HA, Burgess MA, Patel SR, et al. Correlation of computed tomography and positron emission tomography in patients with metastatic gastrointestinal stromal tumor treated at a single institution with imatinib mesylate: proposal of new computed tomography response criteria. J Clin Oncol. 2007 May 1;25(13):1753-9.
26. Gillies RG, Kinahan PE, Hricak H. Radiomics: Images are more than pictures, they are data. Radiology. 2016;278:In Press.
27. Coudert B, Pierga JY, Mouret-Reynier MA, Kerrou K, Ferrero JM, Petit T, et al. Use of [(18)F]-FDG PET to predict response to neoadjuvant trastuzumab and docetaxel in patients with HER2-positive breast cancer, and addition of bevacizumab to neoadjuvant trastuzumab and docetaxel in [(18)F]-FDG PET-predicted non-responders (AVATAXHER): an open-label, randomised phase 2 trial. Lancet Oncol. 2014 Dec;15(13):1493-502.
28. Leen E, Averkiou M, Arditi M, Burns P, Bokor D, Gauthier T, et al. Dynamic contrast enhanced ultrasound assessment of the vascular effects of novel therapeutics in early stage trials. Eur Radiol. 2012 Jul;22(7):1442-50.

**Attribute of IB**	**Impact on technical validation**	**Impact on biological validation**	**Impact on economic viability**	**Biospecimen comparison**	**IB problems and solutions**
1. Imaging devices, agents and software					
Different imaging devices from different vendors are installed in different hospitals	Validation required for all sites	None, if technical validation is performed correctly	Unable to ensure most cost-effective biomarker acquisition (choices limited)<br><br>Priced for radiologic diagnosis not for biomarker use	Biospecimen biomarkers are analyzed using one (or a few identical) in vitro diagnostic devices	Many IBs (e.g. ADC, K^{trans}) give numerically different values on different scanners<br><br>Use of phantoms - exhaustive exercises needed to standardize (e.g. AJCC on staging, RECIST on response, QuIC-ConCePT)<br><br>Development of appropriate phantoms if necessary
Imaging devices may not be designed, maintained or approved for the purpose of measuring the biomarker	Important factors affecting technical validation may be proprietary, obscure, and may change unexpectedly<br><br>Validation required for all sites	None, if technical validation is performed correctly	Resources required for active revalidation of new scanners and upgrades	In vitro diagnostic devices are designed, maintained and approved for specific purpose of measuring the biospecimen biomarker – this substantially reduces risk in technical validation	QIBA seeking regulatory approval for 'off label' biomarkers such as ADC and K^{trans}
Innovation in imaging devices driven by competition to improve picture quality; has unpredictable effect on biomarker measurement	Important factors affecting technical validation may be proprietary, obscure, and may change unexpectedly<br><br>Validation required for all sites	None, if technical validation is performed correctly	Unable to ensure most cost-effective biomarker acquisition	Biospecimen biomarkers have stable platform due to regulatory approval	Parallel imaging changes noise characteristics and may affect measurement (e.g. ADC)<br><br>Gradient improvements affect ADC<br><br>Measure the effect of innovation on biomarker against existing systems
Imaging contrast agent or tracer may not be	Withdrawal of agent in some markets, or failure	May need to be repeated if an alternative tracer is	Priced for radiologic diagnosis not for	Biospecimen biomarkers rarely require	Examples of 'Sinerem', 'Combidex' ferumoxtran,

designed, maintained or approved for the purpose of measuring the biomarker	to develop in certain markets severely limits availability	substituted	biomarker use	administration of a drug substance to the patient	gadomelitol, gadofosveset, and many PET tracers
Non-proprietary software used for many IBs	Validation required for each software version<br><br>Validation required for each software package	May influence biological meaning for biomarker. May need to be repeated if alternative software is substituted	May require proprietary software for translation across second 'Cooksey' translational gap	Biospecimen biomarkers have stable platform due to regulatory approval	Can hinder direct comparison between different centres<br><br>Central analysis hubs for multicentre studies<br><br>Use of phantoms to standardize<br><br>Need for *CE* style approval of software
2. Resources and Staff					
Quality of the IB data dependent on performance of radiologist and technician	Technical validation influenced by each operator<br><br>Mistakes made during scanning cannot be rectified	None, if technical validation is performed correctly	Can lead to increased cost from data exclusion<br><br>Significant ongoing cost for set up, training and retraining	Biospecimen biomarker acquisition often less complex (e.g. phlebotomy) than IBs	Quality of lung/liver biomarkers may depend on control of motion<br><br>Some modalities are very user-dependent (e.g. ultrasound)<br><br>Use highly trained staff, familiar with protocols
Biomarker measurement dictated by workflow in Radiology and Nuclear Medicine departments rather than patient or oncologist needs	May limit throughput and hence recruitment	-	Costs dictated by Radiology and Nuclear Medicine departments	Pathology and Clinical Chemistry departments can accommodate patient and oncologist needs	K^{trans} vary with caffeine intake – timing of scans may be difficult to negotiate, but standardize as much as possible
Many IBs can only be measured on new acquisitions (e.g. new tracer or new sequence is required)	Validation process must begin from scratch	Validation process must begin from scratch	Extremely costly	Validation from pre-banked samples is routine for most biospecimen biomarkers	May take many years to acquire enough new data for adequate statistical power<br><br>Set up and use Image-banks of clinical scan data where possible

					Consortia are acquiring new data for validation (e.g. QuIC-ConCePT for ADC and FLT biomarkers<br><br>Perform multi-centre studies to speed up recruitment
3. Patient Requirements					
Patient commitment required	Takes up patient time<br><br>May involve IA or IV lines<br><br>May be uncomfortable, noisy	May be unrepresentative patients	-	Less patient commitment required for biospecimen biomarkers	Patients may decline recruitment or may drop out of study<br><br>Careful patient selection
4. Tumour Sampling					
IBs are seldom defined analytes	Analytical accuracy is seldom a goal so alternatives must be devised	Biological validation seldom starts from underlying molecular biology ground truth<br><br>Need to agree compelling platform of evidence with all stakeholders	-	Biospecimen biomarkers usually related simply to a defined molecular entity via analytical biochemistry – this is often assumed in validation roadmaps	IBs may be difficult to relate to tissue pathology<br><br>Research required to develop more appropriate tissue pathology standards against which imaging is compared<br><br>Use of PFS/OS
Investigator has control over "sampling" of tumour/disease (both spatially and temporally)	Technical validation needs to be addressed separately for<br>a) biomarker sampling part of lesion (e.g. SUV_{max})<br>b) biomarker sampling entire index lesion (e.g. mean K^{trans})<br>c) biomarker sampling multiple lesions (e.g. metastases)<br>d) biomarker quantifying	Biological validation studies require careful comparison of imaging with single or multiple slice pathology specimens	Whole tumour approaches expected to be effective in capturing disease activity and response (e.g. PFS)	Tumour heterogeneity is a major confound<br><br>Biospecimen biomarkers suffer from confounds at either extremes:<br>a) biomarker from part of lesion (e.g. biopsy)<br>b) biomarker from all disease burden (e.g. circulating biomarker)	Spoilt for choice: must pick small number of primary endpoints that have technical and biological validation<br><br>Whole tumour coverage reduces sampling bias<br><br>ADC, K^{trans} heterogeneity biomarkers may have benefit over average values (e.g. predict outcome)

	entire disease burden (e.g. whole body FDG or DWI)				Evaluate different biology in different lesions
5. Biomarker Validation Landscape					
Little precedent for validation roadmaps in IBs	Little consensus on what constitutes technical validation<br><br>Wildly divergent approaches between different funders, sponsors, and investigators	Little consensus on what constitutes biological validation<br><br>Wildly divergent approaches between different funders, sponsors, and investigators	Acquisition costs, analysis costs and study design make imaging studies expensive but must be addressed<br><br>Outcome studies may be prohibitively expensive, or impossible even in principle	Academic and regulatory roadmaps well-defined for biospecimen biomarkers<br><br>Biological validation on retrospective samples routinely used to construct Kaplan-Meier outcome plots	International consensus being sought from imaging community
