# Supplementary material for: Imaging biomarker roadmap for cancer studies
Source: Nat Rev Clin Oncol. Author manuscript; Available in PMC 2017 Apr 3. (PMC5378302; doi:10.1038/nrclinonc.2016.162)
Supplement: Supplementary information S3 [file NIHMS71926-supplement-Supplementary_information_S3.pdf]

In format provided by O'Connor *et al.* (doi:10.1038/nrclinonc.2016.162)

Supplementary information S3 (box) | Receptor occupancy: pharmacodynamic IB

An early step in the pharmacologic audit trail is to evaluate if the drug engages its target. Displacement of a receptor-targeted PET tracer by the investigational drug provides a specific IB. Aprepitant is a neurokinin-1 (NK_1) receptor antagonist developed for prophylaxis of chemotherapy-induced nausea and vomiting in cancer patients. [^{18}F]SPA-RQC is a NK_1 receptor binding–selective PET tracer with high uptake in the striatum (because it has the highest concentration of NK_1 receptors) and conversely low uptake in the cerebellum. Administration of increasing doses of aprepitant in 16 healthy volunteers in two centres led to an increasing attenuation of striatal [^{18}F]SPA-RQC PET signal allowing receptor occupancy (the IB) to be calculated[1].

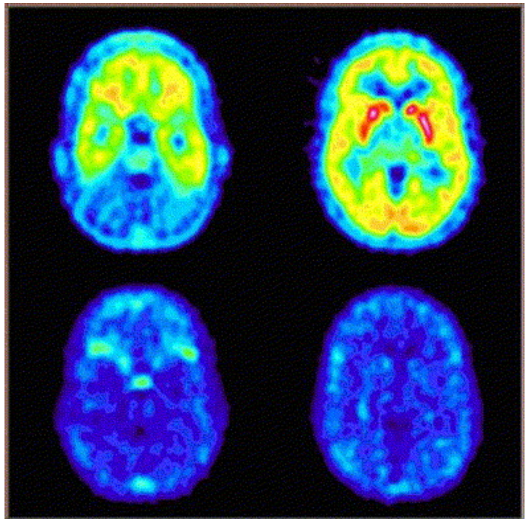

PET image from a subject who received aprepitant 100 mg. Pre-dose (top) and post dose (bottom) PET scans at the level of the cerebellum (left) and striatum (right). Subject number 01, estimated occupancy = 94%. Image reproduced from Bergstrom M. *et al.* Human positron emission tomography studies of brain neurokinin 1 receptor occupancy by aprepitant. *Biol. Psychiatry* **55,**1007–1012 (2004).

Since the % receptor occupancy measurements informed the development of aprepitant, this IB has clearly crossed the translational gap 1 in cancer research. However, it is not expected that this IB would need to cross translational gap 2 to be used daily in healthcare.

References:

[1] Bergstrom M. *et al.* Human positron emission tomography studies of brain neurokinin 1 receptor occupancy by aprepitant. *Biol. Psychiatry* **55,**1007–1012 (2004).
