# Supplementary material for: Imaging biomarker roadmap for cancer studies
Source: Nat Rev Clin Oncol. Author manuscript; Available in PMC 2017 Apr 3. (PMC5378302; doi:10.1038/nrclinonc.2016.162)
Supplement: Supplementary information S4 [file NIHMS71926-supplement-Supplementary_information_S4.pdf]

In format provided by O'Connor *et al.* (doi:10.1038/nrclinonc.2016.162)

Supplementary information S4 (box) | DCE-US AUC: pharmacodynamic and prognostic IB

Dynamic contrast-enhanced ultrasound biomarkers include the area under the curve (AUC), which is an estimate of blood flow. A national initiative in France evaluated a standardized protocol across 19 centres in over 500 patients and demonstrated technical compliance and examination success in 97% cases, following strict criteria for standardized operating procedures[1]. The same data showed reductions in the AUC across a range of tumour primary diagnoses and sites with a variety of antiangiogenic agents. Specifically, a reduction by 90% in this parameter in renal cancer lesions treated with sunitinib related to statistically beneficial time to progression (n=128)[2].

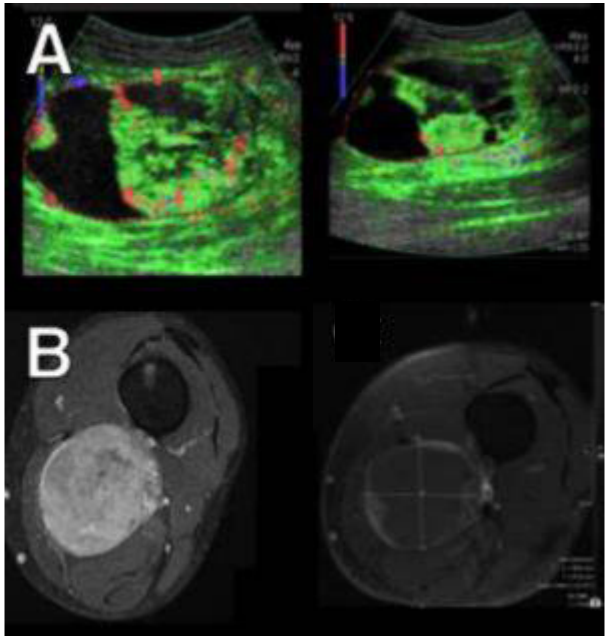

Patient with a thigh synovial sarcoma. (A) the lesion has solid and cystic components. Blood flow is shown in red. The AUC reduces from baseline (left) to pre-surgery (right). (B) Corresponding T_1-weighted MRI contrast-enhanced images.

This IB shows evidence of clinical validation and utility through a large prospective study, so has crossed translational gap 1. Specific application for a drug–tumour pairing seen here with sunitinib and renal cancer begins a qualification process, but this may require evidence of equivalent data in re-test and further technical validation before the IB crosses translational gap 2 and is adopted for routine use in healthcare.

References:

[1] Lassau, N. *et al.* Standardization of dynamic contrast-enhanced ultrasound for the evaluation of antiangiogenic therapies: the French multicenter Support for Innovative and Expensive Techniques Study. *Invest. Radiol.* **47,** 711–716 (2012).

[2] Lassau, N. *et al*. Validation of dynamic contrast-enhanced ultrasound in predicting outcomes of antiangiogenic therapy for solid tumors: the French multicenter support for innovative and expensive techniques study. *Invest. Radiol.* **49,** 794–800 (2014).
