# Supplementary material for: Imaging biomarker roadmap for cancer studies
Source: Nat Rev Clin Oncol. Author manuscript; Available in PMC 2017 Apr 3. (PMC5378302; doi:10.1038/nrclinonc.2016.162)
Supplement: Supplementary information S5 [file NIHMS71926-supplement-Supplementary_information_S5.pdf]

In format provided by O'Connor *et al.* (doi:10.1038/nrclinonc.2016.162)

Supplementary information S5 (box) | Quantitative ADC: putative pharmacodynamic IB

Diffusion-weighted MRI is sensitive to microscopic movement of water molecules and is affected by the cell packing and tissue microstructure. Signals can be quantified to derive apparent diffusion coefficient (ADC). Both diffusion weighted images and ADC are used clinically to detect and characterize cancer[1]. ADC increases in patients and animal tumors that respond to chemotherapy and radiotherapy[2], but changes induced by antivascular agents are inconsistent.

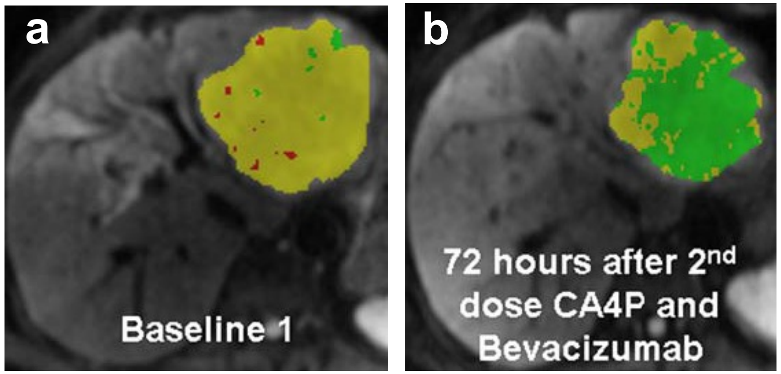

Patient with colorectal liver metastases has (a) relatively homogeneous distribution of ADC values at baseline but (b) after therapy with combination anti-vascular agents shows heterogeneous response with increased ADC in the tumour core consistent with necrosis. Image reproduced from Koh, D.M. *et al.* Reproducibility and changes in the apparent diffusion coefficients of solid tumours treated with combretastatin A4 phosphate and bevacizumab in a two-centre phase I clinical trial. *Eur. Radiol.* **19,** 2728–2738 (2009).

ADC reproducibility is around 10–30% CoV[3]. Technical standardization and quality assurance are being addressed by international consortia [1]. National and international multi-centre trials have shown ADC to be a response, predictive and prognostic biomarker, but at present to our knowledge, no drug development or daily clinical decisions have been made based on quantitative values of ADC (unlike the example of K^{trans}).

References:

[1] Padhani, A.R. *et al.* Diffusion-weighted magnetic resonance imaging as a cancer biomarker: consensus and recommendations. *Neoplasia* **11,** 102–125 (2009).

[2] Henning, E.C., Azuma, C., Sotak, C.H. & Helmer, K.G. Multispectral tissue characterization in a RIF-1 tumor model: monitoring the ADC and T2 responses to single-dose radiotherapy. Part II. *Magn. Reson. Med.* **57,** 513–519 (2007).

[3] Koh, D.M. *et al.* Reproducibility and changes in the apparent diffusion coefficients of solid tumours treated with combretastatin A4 phosphate and bevacizumab in a two-centre phase I clinical trial. *Eur. Radiol.* **19,** 2728–2738 (2009).
