# Supplementary material for: Imaging biomarker roadmap for cancer studies
Source: Nat Rev Clin Oncol. Author manuscript; Available in PMC 2017 Apr 3. (PMC5378302; doi:10.1038/nrclinonc.2016.162)
Supplement: Supplementary information S6 [file NIHMS71926-supplement-Supplementary_information_S6.pdf]

In format provided by O'Connor *et al.* (doi:10.1038/nrclinonc.2016.162)

Supplementary information S6 (box) | MRI ^{13}C-pyruvate k_p: putative pharmacodynamic IB

Hyperpolarized carbon-13 spectroscopic MRI is an emerging technique for measuring tumour metabolism[1]. Pyruvate is a breakdown product of glucose. Injected ^{13}C-labelled pyruvate is exchanged with endogenous tumour lactate (catalysed by lactate dehydrogenase). This exchange can be quantified by a rate constant termed k_p [2], which has been used in animal models to grade tumours and detect early response to therapy [2].

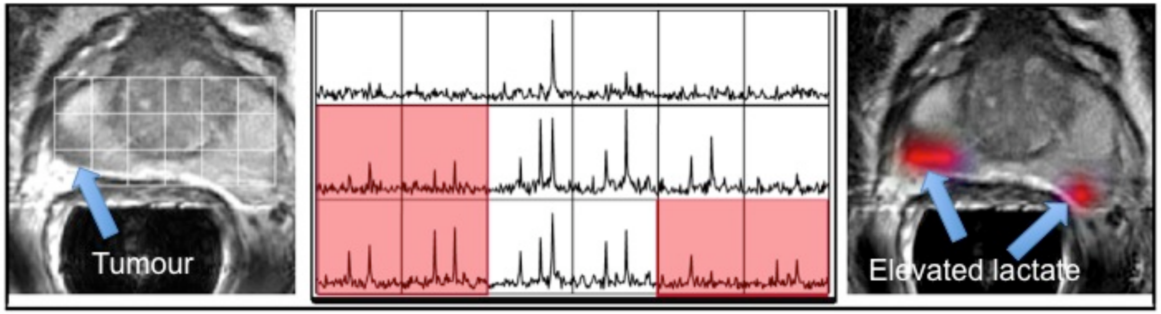

Patient with prostatic cancer has elevated lactate peaks (red voxels) in a known tumour (right peripheral zone) and also in the left peripheral zone (pathological evidence of tumour despite normal anatomical MRI) .

Multiple preclinical studies and a single clinical study[3] have demonstrated potential roles for this IB. Several sites are developing this technology for use in patients, but repeatability, multisite reproducibility and cost effectiveness need to be determined before the IB can cross the first translational gap.

References:

[1] Day, S.E. *et al.* Detecting tumor response to treatment using hyperpolarized (13)C magnetic resonance imaging and spectroscopy. *Nat. Med.* **13,** 1382–1387 (2007)

[2] Bohndiek, S.E. *et al.* Hyperpolarized (13)C spectroscopy detects early changes in tumor vasculature and metabolism after VEGF neutralization. *Cancer Res.* **72**, 854–864 (2012)

[3] Nelson, S.J. *et al.* Metabolic imaging of patients with prostate cancer using hyperpolarized [1-13C]pyruvate. *Sci. Transl. Med.* **5**, 198ra108 (2013).
