# Supplementary material for: Imaging biomarker roadmap for cancer studies
Source: Nat Rev Clin Oncol. Author manuscript; Available in PMC 2017 Apr 3. (PMC5378302; doi:10.1038/nrclinonc.2016.162)
Supplement: Supplementary information S7 [file NIHMS71926-supplement-Supplementary_information_S7.pdf]

Supplementary information S7 (box) | Radiomic signature of heterogeneity: putative prognostic IB

Tumours are biologically heterogeneous and this spatial variation can be imaged[1]. Radiomics quantifies tumour shape, signal intensity/profile, texture and other features to create a signature of the tumour phenotype. This yields 10s to 100s of parameters describing tumour heterogeneity[2], yielding large datasets, comparable to running a gene array. The underlying hypothesis is that important prognostic and/or predictive information is contained within images that is not captured by simple measurement of size or pathophysiology.

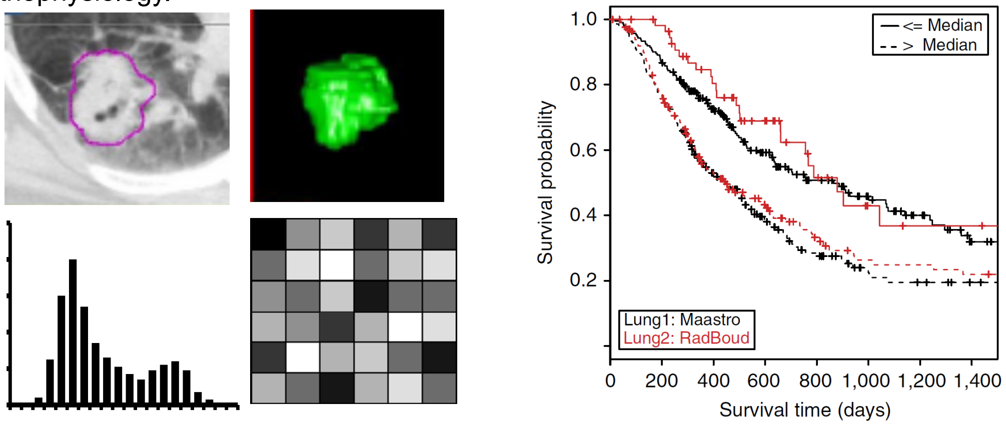

CT demonstrates a tumour in a patient with non-small cell lung carcinoma (NSCLC), from which shape, signal profile, texture and other features are derived. Initial studies suggest that a combination of these features in a signature predict survival in independent NSCLC datasets. Image reproduced from Aerts, H.J. *et al.* Decoding tumour phenotype by noninvasive imaging using a quantitative radiomics approach. *Nat. Commun.* **5,** 4006 (2014).

Radiomic signature has the substantial advantage of being derived from existing clinical data, such as CT or ^{18}F-FDG PET-CT, allowing rapid evaluation of IB versus survival data [2]. This is an emerging IB; it has been applied to a small number of research studies. Further technical validation, biological validation and qualification in other clinical datasets are required before this IB crosses the two translational gaps.

References:

[1] O'Connor, JP. *et al.* Imaging intratumor heterogeneity: role in therapy response, resistance, and clinical outcome. *Clin. Cancer Res.* **21,** 249–257 (2015)

[2] Aerts, H.J. *et al.* Decoding tumour phenotype by noninvasive imaging using a quantitative radiomics approach. *Nat. Commun.* **5,** 4006 (2014).
