# Supplementary material for: Imaging biomarker roadmap for cancer studies
Source: Nat Rev Clin Oncol. Author manuscript; Available in PMC 2017 Apr 3. (PMC5378302; doi:10.1038/nrclinonc.2016.162)
Supplement: Supplementary information S8 [file NIHMS71926-supplement-Supplementary_information_S8.pdf]

In format provided by O'Connor *et al.* (doi:10.1038/nrclinonc.2016.162)

Supplementary information S8 (figure) | Generating biomarkers from an imaging technique

CT, mammography, MRI, PET, SPECT, ultrasound are all **modalities** (classes of imaging), each defined by the underlying physics

Each modality has several **techniques** available

PET techniques include *^{18}F FDG, ^{18}F FMISO/FAZA, ^{89}Zr-bevacizumab*

MRI techniques include *DWI, DCE-MRI, ASL*

Techniques involve tracers, acquisition methods and analysis approaches. They give rise to specific 'objective measurements' or **biomarkers**

Several different IB can be derived from one dataset. Some examples from one whole body DWI scan below include:

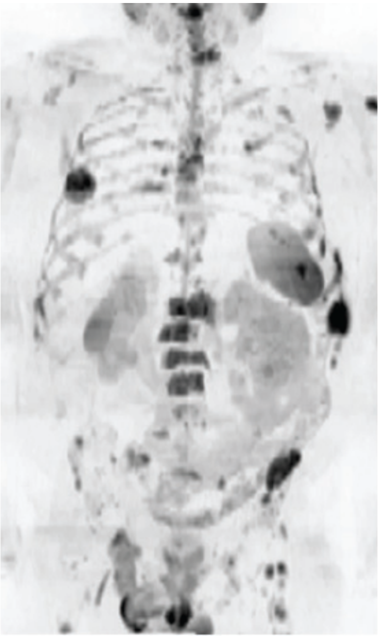

→ **Presence of metastases = M1 (unitless)**

→ **Total tumour burden (mm^3)**

→ **Individual lesion tumour volume (mm^3)**

→ **Median ADC (mm^2/s)**

→ **ADC fractal dimensions (variable units)**
