# Supplementary material for: Imaging biomarker roadmap for cancer studies
Source: Nat Rev Clin Oncol. Author manuscript; Available in PMC 2017 Apr 3. (PMC5378302; doi:10.1038/nrclinonc.2016.162)
Supplement: Supplementary information S9 [file NIHMS71926-supplement-Supplementary_information_S9.pdf]

In format provided by O'Connor *et al.* (doi:10.1038/nrclinonc.2016.162)

Supplementary information S9 (box) | Examples of Bradford Hill criteria for biological validity of IBs

Scientific coherence:	Does the IB faithfully reflect the underlying tumour biology; does it correlate with grade and stage?
Specificity:	Does the IB distinguish responders from nonresponders, or identify those with beneficial or poor progression-free sruvival (PFS) or overall survival (OS)?
Strength of association:	How strong is the association (Hazard Ratio) between the IB and an outcome variable such as PFS, or OS?
Effect gradient:	Does the magnitude of IB change reflect the magnitude of biological change and forecast the magnitude of clinical benefit?
Temporality:	Does the temporal evolution of change in the IB follow the pathological and clinical course as the patient's disease responds, relapses or progress?
Consistency:	Do the strength and specificity of IB and outcome (e.g. survival) have clear effect gradients and temporal patterns that are replicated by different investigators in different settings?
